# Differential psychophysiological interactions of insular subdivisions during varied oropharyngeal swallowing tasks

**DOI:** 10.1002/phy2.239

**Published:** 2014-03-26

**Authors:** Ianessa A. Humbert, Donald G. McLaren

**Affiliations:** 1 Department of Physical Medicine and Rehabilitation, Johns Hopkins School of Medicine, Baltimore, Maryland; 2 Athinoula A. Martinos Center for Biomedical Imaging, Department of Radiology, Massachusetts General Hospital, Charlestown, Massachusetts; 3 Department of Neurology, Massachusetts General Hospital, Boston, Massachusetts; 4 Geriatric Research, Education and Clinical Center, ENRM VA Medical Center, Bedford, Massachusetts; 5 Harvard Medical School, Boston, Massachusetts

**Keywords:** Biofeedback, connectivity, deglutition, gPPI, laterality, psychophysiological interactions, taste

## Abstract

The insula is a highly integrated cortical region both anatomically and functionally. It has been shown to have cognitive, social–emotional, gustatory, and sensorimotor functions. Insular involvement in both normal and abnormal swallowing behavior is well established, yet its functional connectivity is unclear. Studies of context‐dependent connectivity, or the connectivity during different task conditions, have the potential to reveal information about synaptic function of the insula. The goal of this study was to examine the functional connectivity of specific insular regions (ventral anterior, dorsal anterior, and posterior) with distant cortical regions during four swallowing conditions (water, sour, e‐stim, and visual biofeedback) using generalized psychophysiological interactions (gPPI). In 19 healthy adults, we found that the visual biofeedback condition was associated with the most and strongest increases in functional connectivity. The posterior insula/rolandic operculum regions had the largest clusters of increases in functional connectivity, but the ventral anterior insula was functionally connected to a more diverse array of cortical regions. Also, laterality assessments showed left lateralized increases in swallowing functional connectivity. Our results are aligned with reports about the insula's interconnectivity and extensive involvement in multisensory and cognitive tasks.

## Abstract (precis)

The goal of this study was to examine the functional connectivity of specific insular regions (ventral anterior, dorsal anterior, and posterior) with distant cortical regions during four swallowing conditions (water, sour, e‐stim, and visual biofeedback) using generalized psychophysiological interactions (gPPI). In 19 healthy adults, we found that the visual biofeedback condition was associated with the most functional connectivity increases. The posterior insula/rolandic operculum regions had the largest clusters of functional connectivity, but the ventral anterior insula was functionally connected to a more diverse array of cortical regions.

## Introduction

Over the last decade, a series of neural imaging investigations of swallowing have established a swallowing cortical network that involves several regions bilaterally (Hamdy et al. 1999; Martin et al. 2001; Mosier and Bereznaya 2001; Humbert et al. 2009; Malandraki et al. 2010). However, the findings from imaging studies suggest that the swallowing cortical network is broad and not specific to volitional oropharyngeal swallowing alone (Humbert and Robbins 2007; Michou and Hamdy 2009). For instance, cortical processing of volitional oropharyngeal swallowing overlaps with regions involved in nonswallowing tasks (i.e., tongue tapping, lip pursing, and jaw movement; Kern et al. 2001a; Malandraki et al. 2009; Mihai et al. 2013), noncued swallowing (Kern et al. 2001b; Martin et al. 2001), and therapeutic or modified swallowing behaviors (i.e., effortful swallowing; Peck et al. 2010).

Literature reviews and meta‐analyses indicate that the insula is both active and important in swallowing (Humbert and Robbins 2007; Michou and Hamdy 2009; Soros et al. 2009). Additionally, our previous investigations suggest that insular activity is modulated by both the swallowing task (e.g., saliva vs. water) and the sampled population (e.g., young, old, Alzheimer's patients; Humbert et al. 2009, 2010, 2011; Humbert and Joel 2012). Understanding the role of the insula is imperative, given discrepancies in the literature regarding whether damage to a particular insular region is most significant for dysphagia (Daniels and Foundas 1997; Stickler et al. 2003; Riecker et al. 2009; Soros et al. 2011). Previously, we compared the activation patterns of four swallowing conditions using functional magnetic resonance imaging (fMRI): a water bolus, a sour bolus, swallowing with visual biofeedback, and swallowing with surface electrical stimulation (e‐stim). One of the principle findings of this study was that the insula, bilaterally, was the most commonly active cortical region within the swallowing cortical network across all conditions. Amongst the insular areas, the right anterior insula was most active overall across the four conditions (Humbert et al. 2012). Importantly, insular neural activation did not gradually change in response to repeated exposure to the same stimulus over several trials (sensory adaptation or habituation and sensitization). Despite the numerous swallowing fMRI studies, few have focused on the relationship of the insula (functional connectivity) with other regions during swallowing (Mosier and Bereznaya 2001; Lowell et al. 2012; Babaei et al. 2013). Functional connectivity examines how the activity in a chosen seed region is related to spatially distant target regions.

Functional connectivity has previously been examined in volitional swallowing using a principal components analysis, which investigates how the BOLD response amplitudes are similar between brain areas. Using this method, Mosier and Bereznaya (2001) reported that normal volitional swallowing is centrally organized into two parallel circuits including the insula or the cerebellum that connects to sensorimotor, premotor, and parietal modules. More recently, swallowing functional connectivity has also been investigated using seed‐based connectivity, which compare how the significance of temporal correlations between regions changes between conditions. Lowell et al. (2012) examined functional connectivity among cortical regions that integrate motor execution and sensory feedback in the swallowing system in healthy adults. They reported greater clusters of significantly connected voxels from the anterior and posterior insula/rolandic operculum than the other three chosen seed regions; greater functional connectivity was found from the left insula (Lowell et al. 2012). Babaei et al. (2013) examined functional connectivity among three tasks including volitional swallowing, a visual control task, and resting state. The authors reported very high functional connectivity of the anterior and posterior insula within tasks, but comparisons among the tasks revealed no ignificant differences in functional connectivity.

Context‐dependent connectivity, or the connectivity during different task conditions, has the potential to reveal information about synaptic function (Abler et al. 2012). Psychophysiological interactions (PPI), the form of context‐dependent connectivity used in the present analysis, specifically investigate how one brain region (e.g., ventral anterior insula) increases or decreases its relationship with other brain regions under different contexts (Friston et al. 1997; Kim and Horwitz 2008; O'Reilly et al. 2012). Generalized PPI (gPPI; McLaren et al. 2012) assesses how connectivity changes for each task condition relative to the implicit baseline. This method has been shown to be more sensitive and accurate at estimating the pair‐wise connectivity differences between conditions (e.g., novel > repeated) than standard PPI (Cisler et al. 2013) as implemented in SPM5 and SPM8 (McLaren et al. 2012; Cisler et al. 2013). In the present study, the increased accuracy of gPPI allows the detection of subtle differences in connectivity that are related to swallowing biofeedback.

To date, the relationship between insular regions and distant cortical regions of the swallowing network has not been investigated in multiple swallowing tasks. This gap in knowledge has increased speculation about the insula's integrative role in the swallowing cortical network. Thus, the goal of this investigation was to examine the unctional connectivity of specific insular regions (dorsal anterior, ventral anterior, and posterior) with distant cortical regions. The anterior insula was divided into dorsal and ventral portions because recent evidence shows that the dorsal and ventral components are part of different anatomical and functional networks (Mesulam and Mufson 1982a, b; Mufson and Mesulam 1982; Deen et al. 2011; Cerliani et al. 2012; Touroutoglou et al. 2012). The swallowing conditions that were examined include water swallowing, sour bolus swallowing, swallowing with cutaneous electrical stimulation (e‐stim), and swallowing with visual biofeedback. The sour, visual biofeedback, and e‐stim conditions were chosen because, compared to water swallowing, they are known to alter swallowing biomechanics and neural processing (Ding et al. 2003; Crary et al. 2004; Gallas et al. 2010; Humbert et al. 2012). It is unknown whether these sensory modalities can alter functional connectivity of swallowing. To more fully understand the functional connectivity of swallowing, we also investigated hemispheric laterality and gradual changes across consecutive swallowing trials.

We hypothesized that functional connectivity patterns would be consistent with the observed task activity during each swallowing task. Specifically, sour bolus swallowing would have greater connectivity in the ventral anterior insula, due to its role in taste processing (Veldhuizen and Small 2011; Veldhuizen et al. 2011a, b). We predicted that swallowing with cutaneous electrical stimulation would have greater connectivity in the dorsal anterior insula, given our previous findings and those of others (Alkire et al. 2004; Zarate et al. 2010; Humbert et al. 2012). We expected visual biofeedback to have the most connectivity overall, as it was most activated relative to other conditions in our prior study (Humbert et al. 2012). Regarding laterality, we expect that our swallowing conditions will show increased functional connectivity in the left hemisphere, consistent with Lowell et al. (2012). Although insular activation did not show evidence of adaptation with functional MRI (Humbert et al. 2012), we will also test whether gradually increasing (sensitization) or decreasing (habituation) functional connectivity occurs. We predict there will be no habituation or sensitization of the signal, similar to our previous findings. Results from this investigation may identify potential neural networks that are potentially disrupted and contribute to individuals with dysphagia.

## Methods

We conducted an event‐related functional magnetic resonance imaging (fMRI) experiment of swallowing in nineteen healthy adults (mean age 46.6 years SD ± 22.4), of which we previously reported the evoked task effects or BOLD response (Humbert et al. 2012). No participant had a history of swallowing, speech, or cognitive disorders, or any other chronic medical condition. All participants provided written informed consent to participate in this study, which was approved by the Institutional Review Board of the Johns Hopkins Medical Institute and in accordance with the Declaration of Helsinki.

### MRI protocol

#### Functional MRI acquisition

All MR imaging was acquired with a 3T Phillips MRI scanner with an 8‐channel head coil. Using multislice 2D SENSE T2* gradient‐echo, echo planar imaging (EPI) pulse sequence, functional images were obtained in the axial plane. Higher order shimming was applied to the static magnetic field (B0). The EPI parameters were as follows: echo time (TE) = 30 ms; repetition time (TR) = 2000 ms; flip angle (FA) = 75°; matrix = 80 × 80; FOV = 240 × 240 mm; SENSE factor = 2; 37 sequential/interleaved slices each 3 mm thick with a 1 mm gap between them. This protocol acquired 194 temporal whole‐brain image volumes, with the first five volumes being discarded to ensure magnetization equilibrium. Additionally, high‐resolution T1‐weighted structural imaging utilized a magnetization‐prepared rapid acquisition with gradient echo (MP‐RAGE) sequence with the following parameters: TE = 3.7 ms; TR = 8.0 ms; inversion time = 843 ms; FOV = 256 × 200 mm; FA = 8°; matrix = 256 × 200; SENSE factor = 2; and 200 coronal slices that were 1.0 mm thick. Anatomical scans were used as an intermediate for spatial normalization of functional scans, for clinical over‐reads to detect abnormalities and exclude ineligible participants.

#### Task design

This study involved 4 runs with 80 swallows using the same technique previously published (Humbert et al. 2012). Five milliliters of room‐temperature liquid was infused directly onto the anterior‐mid region of the tongue via plastic tubing that was dispensed by a MR‐safe injector (Spectris Solaris^®^, Medrad). Each run consisted of a single swallowing condition with 20 swallows. The four conditions were: distilled water, sour liquid, distilled water with cutaneous electrical stimulation (e‐stim), and distilled water with visual biofeedback of swallowing. The order of the four runs was randomized across participants. Sour water and distilled water were infused with separate tubing to avoid taste contamination. Participants were instructed to swallow once they felt that the liquid had completely entered their mouths and the interstimulus interval was 18 s for all swallows. Task compliance was monitored with an oral pressure system that consisted of a water‐filled tube that extended from the oral cavity to a transducer, which measured fluid displacement with each swallow. This pressure transducer only detects pressure differences in the oral cavity and no pressure changes could be detected by pushing directly on the small tubes in the mouth. To remove any residual sour taste on the tongue, a wash out period followed the taste run.

#### Swallowing conditions

##### Water

The distilled water run was the control condition.

##### Sour

The taste condition included a sour bolus (citric acid USP 0.65 g/100 mL distilled water, odorless).

##### E‐stim

Cutaneous electrical stimulation was administered to the anterior neck with two adhesive surface electrodes (silver/silver chloride Ambu®; skin contact size 28 × 20 mm in diameter) located on either side of the larynx and approximately one‐inch apart, as determined by palpation. The location was chosen based on improvements in swallowing in individuals with dysphagia (Gallas et al. 2007, 2010; Ludlow et al. 2007). E‐stim was only administered during swallowing and only at a (low) sensory level (approximately 2 s per swallow) that does not recruit muscle activity. Thus, the e‐stim condition is truly the combined effect of swallowing and electrical stimulation as they occurred simultaneously. As swallowing is a complex movement, a control condition consisting of another movement paired with electrical stimulation was not possible to achieve. Each participant determined the stimulation intensity, whereby they indicated when the stimulation was felt (typically a prickly sensation), but without a muscle contraction. The first author has previously administered sensory‐level and motor‐ or muscle contraction‐level stimulation to the skin overlying the larynx (Humbert et al. 2006; Ludlow et al. 2007).

##### Visual biofeedback

**Figure 1.**
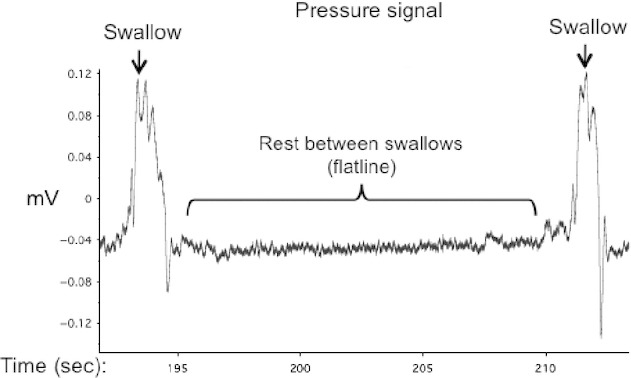
Visual Biofeedback Signal. The swallowing signal shown to participants during the visual biofeedback condition. Swallows are seen as changes in amplitude from rest (or flatline) periods between swallow trials.

The visual biofeedback condition consisted of continuously displaying the signal from the oral pressure‐monitoring system to the participant. The pressure‐monitoring system allowed real‐time monitoring of oral pressure changes (by the investigator) and presentation to the participant occurred simultaneously. Participants were told to swallow normally and to view the signal that represented swallowing behavior. Since signal amplitude changes representative of actual swallowing occurred only during swallowing, the periods between swallowing in this condition displayed a flat‐lined signal, unlike EMG, which can be overly sensitive to small tongue movements between swallowing events (Yeates et al. 2010; Fig. 1). Thus, swallowing with visual biofeedback (signal amplitude changes) was implicitly contrasted against visual biofeedback (flat‐lined signal). The other conditions occurred with a white glare seen through the mirror, to control for effects of light separate from the oral pressure signal information. All participants could clearly see the signal without adjusting their head position.

The interpretation of the conditions is as follows. Water swallowing is interpreted as the control condition, which will be compared to no swallowing and to the three other conditions. Sour swallowing is interpreted as the control effect of swallowing with added gustatory input. E‐stim is interpreted as the control effect of swallowing with added electrical stimulation input. Visual biofeedback is interpreted as the control effect of swallowing with added visual input. The general effect of visual input (white glare from the projector) is removed from the visual biofeedback condition as it was present during each condition.

### Image preprocessing

All functional images were preprocessed with Statistical Parametric Mapping (SPM5, Wellcome Department of Imaging Neuroscience, University College London, UK). Functional images went through the following processing steps: (1) slice‐time correction and (2) motion correction. Then the T1‐weighted image was coregistered with the functional images. Next, “unified segmentation” was performed on the T1‐weighted images to determine the normalization parameters needed to warp data from native to MNI space. These parameters were then used to warp the functional images to MNI space and sampled to 2 mm isotropic voxels. The warped functional images were then smoothed with 6 mm FWHM Gaussian kernel.

### First‐level analyses: task activity

First‐level analyses of the time series data were performed for individual participants using a general linear model. Swallow onset times for each condition were obtained directly using the oral pressure signals. The vectors of onset for each condition were convolved with the canonical hemodynamic response function (HRF) to construct the statistical model, resulting in a 4‐column design matrix. In addition, the six motion parameters obtained from motion correction was added for each session in the design matrix to account for spin history artifacts associated with motion. Time points with higher than 3 mm translational or 2 degrees rotational differential motion were removed using stick regressors. The general linear model removed the low frequencies with a 128 s high‐pass filter. Additionally, a parametric modulator for trial number was also included to allow for the investigation of habituation or sensitization. Group effects from these models were reported previously (Humbert et al. 2012).

### First‐level analyses: generalized psychophysiological interactions

Generalized Psychophysiological Interactions (gPPI), based on their improved sensitivity and specificity in detecting connectivity effects (McLaren et al. 2012), were used to compute the context‐dependent connectivity of six insular seed regions. gPPI models were created and estimated using the publicly available gPPI Toolbox (http://www.nitrc.org/projects/gppi).

The gPPI Toolbox uses the following equation to estimate the PPI effects:



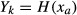





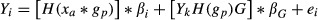



where ***H*** is the HRF in Toeplitz matrix form; **Y**_**k**_ is the BOLD signal observed in the seed region; **x**_**a**_ is the estimated neural activity from the BOLD signal in the seed region (Gitelman et al. 2003); **Y**_**i**_ is the BOLD signal observed at each voxel in the brain; ***β***_**i**_ is a matrix of the beta estimates of the psychophysiological interaction terms; ***β***_**G**_ is a matrix of the beta estimates of the seed region BOLD signal (**Y**_**k**_), covariates of no interest (**G**), and convolution of psychological vectors *H*(**g**_**p**_); **e**_**i**_ is a vector of the residuals of model; and **g**_**p**_ is a matrix of N columns, where N is the number of conditions in the experiment and formed by separating the time when the conditions are present into separate columns. Additionally, if the time when the condition is present is weighted by a parametric modulator, such as swallow number, gPPI can also assess parametric changes in connectivity within a condition.

**Figure 2.**
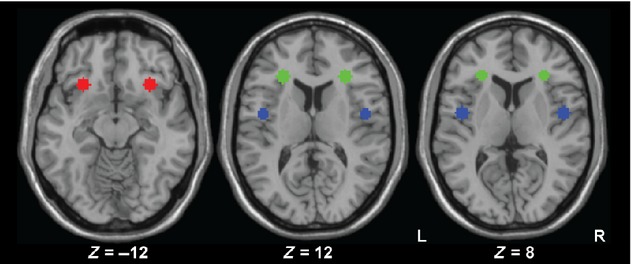
Psychophysiological Seed Regions shown on axial slices from single subject in MNI space. Red regions are the ventral anterior insular seed regions centered at *Z* = −12. Green regions are the dorsal anterior insular seed regions centered at *Z* = 12. Blue regions are the posterior insular seed regions centered at *Z* = 8. R; right hemisphere; L, left hemisphere.

For the present analysis, we chose six seed locations a priori to understand both the laterality and anterior–posterior effects of the swallowing on insular connectivity (Fig. 2). Each seed region was defined as 6‐millimeters around the center of group peak activity maps or the contralateral MNI coordinate. The six insular regions were: (1) left ventral anterior insula (contralateral voxel of the group sour activation peak, MNI:−30, 22, −12); (2) left dorsal anterior insula (peak voxel from the group e‐stim activation, MNI: −28, 30, 12); (3) left posterior insula/rolandic operculum (peak voxel from the group visual biofeedback activation, MNI: −46, −4, 8); (4) right ventral anterior insula (peak voxel from the group sour activation, MNI: 30, 22, −12); (5) right dorsal anterior insula (contralateral voxel of the group e‐stim activation peak, MNI: 28, 30, 12); and (6) right posterior insula/rolandic operculum (contralateral voxel of the group visual biofeedback activation peak, MNI: 46, −4, 8). Peak voxels from the group maps and their associated spheres were labeled based on the Automated Anatomical Labeling atlas (Tzourio‐Mazoyer et al. 2002). We chose to select the contralateral voxel as the seed center for testing laterality as it is entirely objective and closely matches the same anatomical area.

### Second‐level analyses: gPPI

Group analysis for the effects of swallowing was analyzed with GLM Flex (http://mrtools.mgh.harvard.edu/index.php/Main_Page) that allows for the analysis of both within–subject and between–subject effects in the same repeated‐measures ANOVA, a feature not available in SPM8. Age group (old and young) added as a factor of no interest in the models. Separate models were run for the swallow and habituation/sensitization effects in each seed region. Thus, there were 12 repeated‐measure ANOVAs estimated in this analysis. Comparison from these models included: (1) comparison of each condition to no swallowing (condition‐specific effects) and (2) pair‐wise comparisons of conditions (condition comparison effects).

To correct for multiple comparisons, we determined, using 3dClustSim (Analysis of Functional NeuroImages), that a threshold of *P* < 0.005 in at least 51 contiguous voxels (408 mm^3^) yields a cluster corrected *P* < 0.05. We use this threshold for reporting all voxel‐wise findings. Each gPPI seed region analysis can be considered to be an independent analysis, as we do not directly compare the gPPI contrasts between seeds at the voxel or cluster level. Thus, a correction for the number of seed regions is not needed in this study. As we wanted to establish the gPPI effects for each swallow biofeedback separately and provide evidence for selecting future swallow biofeedback conditions in future studies, we did not correct for the number of conditions.

Additionally, we report the probability of finding the number of significant clusters for each region and condition, which is referred to at the set‐level *P*‐value, using random field theory in SPM8.

### Third‐level analyses: spatial distribution of connectivity

**Figure 3.**
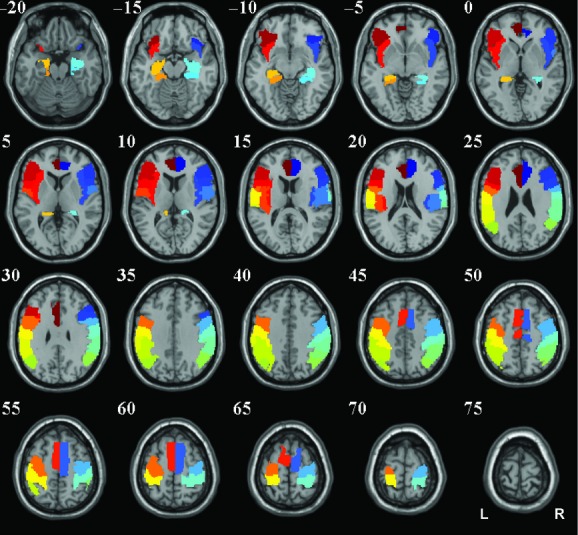
Swallow network shown on axial slices from a single subject in MNI space. All regions are from the automated anatomical label atlas (Tzourio‐Mazoyer et al. 2002). From anterior to posterior: Anterior cingulate cortex; inferior frontal gyrus – orbital part; inferior frontal gyrus – pars triangularis; inferior frontal operculum; insula; supplementary motor area; rolandic operculum; precentral gyrus; parahippocampal gyrus; hippocampus; postcentral gyrus; supramarginal gyrus; inferior parietal lobule; angular gyrus. R; right hemisphere; L, left hemisphere.

**Figure 4.**
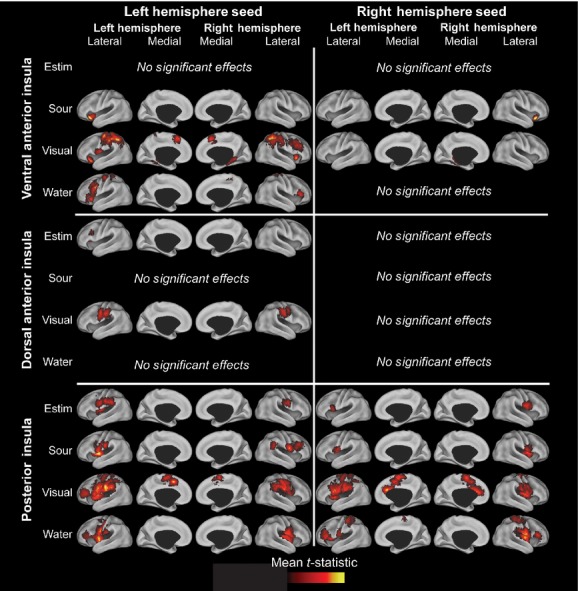
Cortical Surface Renderings of Significant Connectivity Changes During Swallowing. This image depicts the cortical rendering of significant regions (*P* < 0.05 cluster corrected) that were differentially connected to the seed (insula) during swallowing. Cortical surface renderings on the PALS CARET surface (Van Essen 2005) for each swallowing task > implicit baseline (no swallowing) contrast for the ventral anterior insula (top row), dorsal anterior insula (middle row), or posterior insula seeds (bottom row). The left column has the left hemisphere seeds, while the right column has the right hemisphere seeds. Multiple comparison corrected maps (*P* < 0.005 in at least 51 contiguous voxels, cluster corrected *P* < 0.05) were projected to the surface using multifiducial mapping with the strongest voxel within 2.5 mm of each surface node.

**Figure 5.**
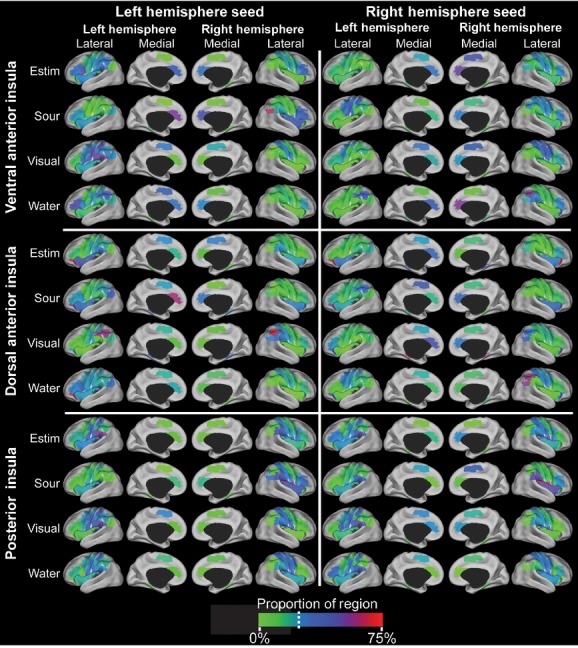
Spatial Distributions. This image depicts the cortical rendering of the proportion of each region that in the top 25% of differentially connected voxels to the seed (insula) during swallowing. The spatial distributions (Figure 7) were back‐projected to the AAL regions that were used to create the distributions and then projected to the PALS CARET surface (Van Essen 2005) using average fiducial mapping and the enclosing voxel. The value in each region represents the proportion of the region that contained voxels that were in the top 25% of all voxels in the mask. The dashed line indicates the color of regions if the voxels were randomly distributed in the mask. If all regions were close to this color, then the distribution would be random or spurious. Based on chi‐square tests, none of the insular seeds or conditions had a random distribution. All of them had some preference for at least a few regions.

Using 28^1^ regions identified as being involved in swallowing (Fig. 3; Humbert and Robbins 2007; Michou and Hamdy 2009; Malandraki et al. 2011), we counted the number of voxels in each region that were in the top 25% of all connectivity changes, based on the t‐statistics, in the swallowing network. The regions were selected from the automated anatomical labeling atlas (Tzourio‐Mazoyer et al. 2002). This was repeated for each condition and seed region. While the choice of 25% is arbitrary, other studies have tested multiple thresholds (i.e., 5% and 1%) and found no differences in the interpretation of the results (Fig. 4). As the null hypothesis is that all spatial distributions are the same, the only detriment of choosing different thresholds would be to potential for false negatives – where the conclusion is that the spatial distributions are the same. Selecting a specific number of voxels from each analysis and correcting for network size allows the comparison of spatial distributions. Figures 5 and 7 show the proportion of voxels in each region that are in the top 25% of all connectivity changes in the mask. If 25% of the connectivity changes in each region fell within the top 25% of all connectivity changes (Figs. 5, 7), then the connectivity increases could be considered random or spurious. When they differ from containing equal proportions of the top 25% of the connectivity changes (e.g., 25%), we conclude that there is regional specificity to the connectivity increases. Using chi‐squared tests, we assessed whether these patterns were different than a uniform distribution, different between feedback conditions, different between seeds, and different between hemispheres (e.g., was the left seed differently connected to the left or right hemisphere). To correct for multiple comparisons, significant results are reported for comparisons at a bonferroni corrected threshold of *P* < 0.05. These metrics enable interpretations about the regional and feedback specificity of swallowing.

### Third‐level analysis: laterality of connectivity

Laterality was assessed using three analyses. First, we \compared the left and right hemisphere PPI distributions for each seed‐task pairings using a chi‐squared test (described above). Second, we computed the laterality index as in Lowell et al. (2012). This approach compares the volumes of significant PPI effects for the left and right hemispheres using the following equation:
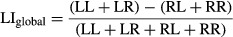


Where LL is the left seed, left hemisphere connectivity, LR is the left seed, right hemisphere connectivity, RL is the right seed, left hemispheres connectivity, and RR is the is the right seed, right hemisphere. Additionally, we assessed the laterality separately for the left and right hemisphere:



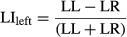





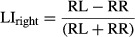



LIs were computed for the voxels within clusters with a significant PPI effect (*P* < 0.005 in at least 51 voxels). Positive LI values reflect a left hemisphere asymmetry, while negative LI values reflect a right hemisphere asymmetry. A variety of thresholds have been used to classify an effect as lateralized or not lateralized ranging from 0.1 to 0.3 (Lowell et al. 2012). In the present study, we used a threshold of 0.3 to classify a region as lateralized.

## Results

All 19 participants completed this study without adverse events.

### Summary of activation findings from Humbert and Joel (2012)

**Figure 6.**
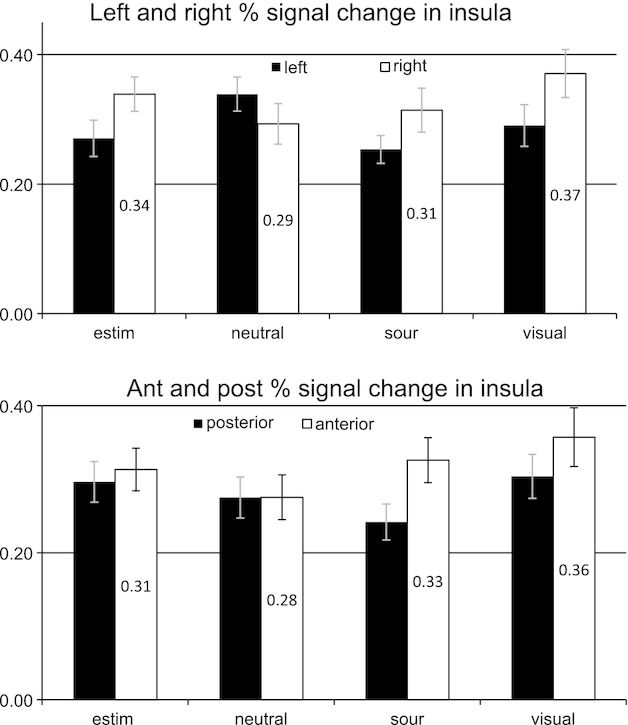
BOLD Signal in Insula. Mean peak percent BOLD signal change within the insula displaying left–right and anterior–posterior differences for each condition (originally published in NeuroImage 59(2): 1485–1490).

Humbert et al. (2012) differentiated signal within the insula by left and right as well as the anterior and posterior insula components across the same four conditions (Fig. 6). Compared to the water condition, the findings reveal greater activation in the right anterior insula for the three sensory conditions. Conversely, the water signal had somewhat more signal in the left insula, but balanced signal between anterior and posterior regions.

### Summary of connectivity findings

Overall, significant functional connectivity changes (*P* < 0.05 cluster corrected) between the insula and distant regions of the swallowing cortical network were identified during swallowing (see cortical renderings and spatial distributions for all conditions and insular regions in Figs. 4, 5). When the spatial distributions are different between regions and greater than 25% of a region (blue to red), then the connectivity increases have regional specificity and are not spurious or random. The posterior insula/rolandic operculum had more significant functional connectivity increases across the four swallowing tasks (9720 voxels, 49 clusters, set‐level *P*‐value range: 0–0.06) than the ventral anterior insula (3988 voxels, 22 clusters, set‐level *P*‐value range: 0–1) and the dorsal anterior insular region (572 voxels, 4 clusters, set‐level *P*‐value range: 4.52 × 10^−5^−1). Swallows during visual biofeedback had the most significant functional connectivity increases across the insula (9460 voxels, 39 clusters, set‐level *P*‐value range: 0–1), followed by water swallowing (3066 voxels, 21 clusters, set‐level *P*‐value range: 1.11 × 10^−16^−1), sour liquid swallowing (1015 voxels, 9 clusters, set‐level *P*‐value range: 1.07 × 10^−10^−1), then swallowing with e‐stim (739 voxels, 6 clusters, set‐level *P*‐value range: 4.52 × 10^−5^−1). Only one cluster of 63 voxels had significant functional connectivity decreases during sour liquid swallowing, thus the results will focus on the functional connectivity increases. Tables 5–10 provide details of cortical regions, set‐level *P*‐values, statistical significance for each peak voxel, and MNI coordinates for all findings.

### Condition‐specific effects

#### Visual biofeedback

The visual biofeedback condition elicited the most significant functional connectivity increases (cluster corrected *P* < 0.05) between the insular regions and spatially distant regions associated with swallowing. During this task, multiple brain regions had increased connectivity within the frontal and parietal lobes, limbic system and the insula. The visual biofeedback condition was associated with significantly increased functional connectivity between the insula and the limbic system, including the hippocampus, parahippocampus, and anterior cingulate cortex compared to the other swallowing conditions. Additionally, the significantly increased connectivity in these regions was only found during the visual biofeedback condition and was significantly greater than the increases in connectivity during other conditions. Swallowing with visual biofeedback was associated with the most diverse pattern of connectivity increases for the ventral anterior insula, which had increased connectivity to all of the selected brain regions except ACC. The posterior insula/rolandic operculum had the greatest extent of significant increases in functional connectivity (5648 voxels, 22 clusters, set‐level *P* = 0 and 1.11 × 10^−16^, for the right and left hemispheres, respectively), while the dorsal anterior insula had the least amount of significant increases in connectivity (519 voxels, 3 clusters in the left seed, set‐level *P* = 4.52 × 10^−5^) change compared to not swallowing (Fig. 4, Table 1).

**Table 1.** Condition‐specific Effects. This table shows regions with significant clusters (*P* < 0.05 cluster corrected threshold) for each swallowing condition (V = visual, E = e‐stim, S = sour, W = water) compared to baseline. Significant regions are the distant brain regions for which significant functional connectivity was found relative to the insular seed region (dorsal anterior, ventral anterior, and posterior).

	Dorsal anterior insula				Ventral anterior insula				Posterior insula			
Frontal												
M1	V		S		V	W			V	W	S	E
IFG Tri					V	W				W	S	
IFG Oper				E	V	W			V			
IFG Orb					V	W	S					
SMA					V				V	W		
Parietal												
S1	V		S		V	W			V	W	S	E
IPG	V				V				V			
SMG	V				V				V	W	S	E
Angular					V				V			
Limbic												
ACC									V			
Hippocampus					V							
Parahipp					V							
Insula					V	W	S		V	W	S	

M1, precentral gyrus, IFG Tri, inferior frontal gyrus triangularis, IFG oper, inferior frontal gyrus opercularis, SMA, supporting motor area, S1, postcentral gyrus, IPG, inferior parietal gyrus, SMG, supramarginal gyrus, ACC, anterior cingulate cortex, RO, Rolandic operculum.

#### Water

The water condition had significantly increased functional connectivity (cluster corrected *P* < 0.05) from the ventral anterior and posterior insular/rolandic operculum regions, but not the dorsal anterior insula. During water swallowing, increased functional connectivity was observed between the ventral anterior insula and the posterior insular/rolandic operculum regions and M1, IFG triangularis, S1, the rolandic operculum and the insula. The significant voxels did not overlap between seed regions; however they were not significantly different from each other indicating that the connectivity in these regions was similar in magnitude.

#### Sour

Functional connectivity was significantly increased (cluster corrected *P* < 0.05) between each of the three insular regions and spatially distant regions during sour liquid swallows, as with the visual biofeedback condition, albeit with far fewer voxels. The dorsal anterior insula had increased connectivity with M1 and S1, while the ventral anterior insula had increased connectivity with the orbital region of the IFG and the insula. When swallowing sour liquid, functional connectivity increases were most prevalent between the posterior insula/rolandic operculum and distant regions, including M1, IFG triangularis, S1, SMG, the rolandic operculum and the insula.

#### E‐stim

The e‐stim condition had the least amount of significant functional connectivity increases of the four conditions (cluster corrected *P* < 0.05). Functional connectivity increases were observed between the dorsal anterior insula and orbital part of the IFG, M1, S1, the rolandic operculum and the insula. The e‐stim condition also had increases between the posterior insula and IFO, M1, S1, SMG, the rolandic operculum and the insula.

### Condition comparison effects

Findings for condition contrasts revealed that the visual biofeedback swallowing condition had significantly greater increases in functional connectivity (cluster corrected *P* < 0.05) when contrasted with other swallowing conditions, overall. In particular, the visual condition had greater functional connectivity increases than e‐stim and sour conditions in all three insular regions and contrasted with water in the ventral and dorsal anterior insular regions. No condition had greater functional connectivity increases than the visual biofeedback condition at a cluster corrected *P* < 0.05. Second to the visual condition, water swallowing had greater functional connectivity increases than sour and e‐stim in both the ventral anterior (insula bilaterally) and the right posterior insula/rolandic operculum. Swallowing with e‐stim had greater functional connectivity increases than sour (left dorsal anterior insula and right posterior insula) and water swallowing (right dorsal anterior insula). In small clusters, sour swallows had greater increases than water swallows with the right dorsal anterior insula and greater increases than e‐stim with the left posterior insula (cluster corrected *P* < 0.05; Table 2).

**Table 2.** Condition Contrasts. The summary of condition contrasts by insular seed region separated by hemisphere. The number of voxels in significant clusters (*P* < 0.05 cluster corrected) for each pair‐wise condition comparison and number of cortical regions showing significant clusters for each pair‐wise condition comparison is shown.

Insular regions	L. VAI		R. VAI		L. DAI		R. DAI		L. PI		R. PI	
	Cluster size	No. of regions	Cluster size	No. of regions	Cluster size	No. of regions	Cluster size	No. of regions	Cluster size	No. of regions	Cluster size	No. of regions
Visual > E‐stim	2206	9	301	2			195	1	68	1		
Visual > Sour	1171	7			1900	7			130	2	234	3
Visual > Water	119	1	69	2			771	4				
Water > E‐stim	520	5	288	1							80	1
Water > Sour	133	4	54	1							362	3
Water > Visual												
E‐stim > Sour					417	3					91	1
E‐stim > Water							120	2				
E‐stim > Visual												
Sour > Water							54	1				
Sour > E‐stim									51	1		
Sour > Visual												

L. VAI, left ventral anterior insula; R. VAI, right ventral anterior insula; L. DAI, left dorsal anterior insula; R. DAI, right dorsal anterior insula; L. PI, left posterior insula; R. PI, right posterior insula.

### Insular regions

#### Overall

All three insular regions had significant connectivity increases with distant cortical regions during all four conditions (cluster corrected *P* < 0.05). The posterior insula and ventral anterior insula were functionally connected bilaterally. Our laterality assessments indicated that swallowing functional connectivity with the insula as a seed region is left lateralized and increases in connectivity are more ipsilateral than contralateral (Figs. 4, 5 and Table 3).

**Table 3.** Laterality of Connectivity Changes During Oropharyngeal Swallowing by Seed Region and Task. The global and hemispheric laterality indices for each region. Positive LI values reflect a left hemisphere asymmetry, while negative LI values reflect a right hemisphere asymmetry. Indices greater than 0.3 or less than −0.3 indicate lateralization (bolded and italicized).

	Global LI	Left LI	Right LI
Ventral Anterior Insula			
*E‐stim*	NA	NA	NA
*Sour*	0.04	***1.00***	***−1.00***
*Visual*	***0.95***	0.11	***1.00***
*Water*	***1.00***	***0.56***	NA
*Average*	***0.71***	***0.70***	−0.17
Dorsal Anterior Insula			
*E‐stim*	***1.00***	***1.00***	NA
*Sour*	NA	NA	NA
*Visual*	***1.00***	***0.12***	NA
*Water*	NA	NA	NA
*Average*	***1.00***	***0.52***	NA
Left Posterior Insula			
*E‐stim*	***0.37***	***0.66***	−***0.48***
*Sour*	***0.43***	0.30	−***0.55***
*Visual*	0.10	***0.41***	0.28
*Water*	−0.14	***0.30***	−0.19
*Average*	−0.05	0.05	0.06

LI, Laterality Index; NA, values could not be computed because there were no significant voxels.

#### Ventral anterior insula

**Figure 7.**
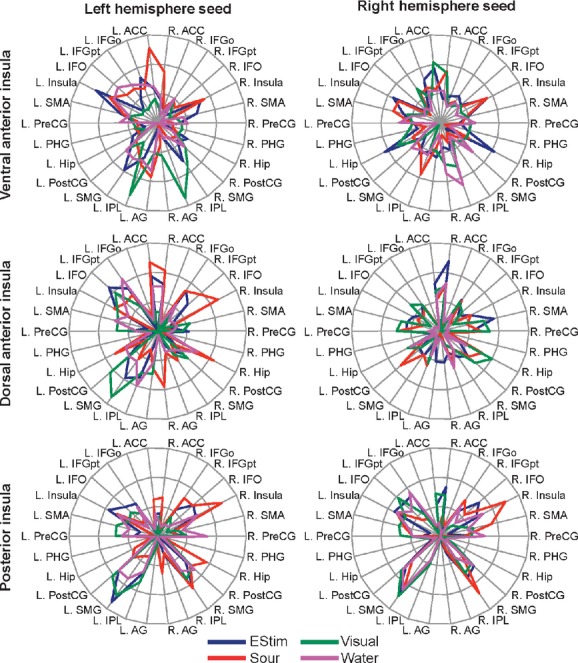
Spatial distributions for each swallow>baseline PPI effect by swallowing region. The plots are based on the top 25% of voxels showing an effect with the left ventral anterior insular seed (top left), the left dorsal anterior insular seed (middle left), the left posterior insular seed (bottom left), the right ventral anterior insular seed (top right), the right dorsal anterior insular seed (middle right), and the right posterior insular seed (bottom right). Rings are drawn at 25%, 50%, and 75% of a region. If the PPI effects were entirely random, the lines would follow the 25% ring. Abbreviations: EStim, swallow with cutaneous electrical stimulation; Sour, sour liquid swallows; Visual, swallow with visual biofeedback; L., left; R., right; ACC, anterior cingulate cortex; IFGo, inferior frontal gyrus ‐ orbital part; IFGpt, inferior frontal gyrus ‐ pars triangularis; IFO, inferior frontal operculum; SMA, supplementary motor area; PreCG, precentral gyrus; PHG, parahippocampal gyrus; Hip, hippocampus; PostCG, postcentral gyrus; SMG, supramarginal gyrus; IPL, inferior parietal lobule; and AG, angular gyrus.

The ventral anterior insula had the most diverse connectivity, as significant connectivity increases were found for all distant cortical regions except the anterior cingulate cortex across the four swallowing conditions (Table 1, cluster corrected *P* < 0.05). The global laterality index was significantly left lateralized to the left hemisphere for visual biofeedback and water swallows (Table 3). The left ventral anterior insula had significant connectivity increases to the left hemisphere for sour swallows, visual biofeedback, and water swallows. The right ventral anterior insula had significant connectivity increases to the right hemisphere for sour swallows and connectivity increases to the left hemisphere for visual biofeedback swallows. The analysis of spatial distribution of connectivity increases revealed that the left and right ventral anterior regions have significantly different patterns of connectivity increases and that the patterns are lateralized to the left hemisphere (Figs. 4, 5, Tables 3 and 4). Additionally, each task pattern was significantly different (Figs. 5, 7, Tables 2 and 4). Both the laterality indices and analyses of the distributed patterns indicate that the ventral anterior insula is significantly left lateralized and has increased connectivity with the left hemisphere. Other than the visual biofeedback condition (right insula to left hemisphere), connectivity increases were generally ipsilateral (Table 3).

**Table 4.** Differences in the Spatial Distribution of PPI Effects During Oropharyngeal Swallowing by Seed Region and Task. Values are the chi‐squared statistics for seed region or condition pair‐wise comparisons of the voxel distribution for the top 25% of voxels of the contrast. Any statistic above 59.1 would be significant at an alpha of 0.01 after correcting for multiple comparisons. Thus, all comparisons were significant.

		E‐stim	Sour	Visual	Water			E‐stim	Sour	Visual	Water
Left Ventral Anterior Insula	E‐stim		2147.00	3297.66	2037.55	Right Ventral Anterior Insula	E‐stim		1255.99	1821.45	3192.14
	Sour			3462.37	2240.56		Sour			1881.67	2458.09
	Visual				3242.90		Visual				1834.57
	Water						Water				
Left Dorsal Anterior Insula	E‐stim		3776.51	2651.39	1716.44	Right Dorsal Anterior Insula	E‐stim		1923.29	1804.63	3069.31
	Sour			7318.95	3743.94		Sour			1481.97	2555.45
	Visual				2832.20		Visual				3183.26
	Water						Water				
Left Posterior Insula	E‐stim		2199.72	1273.22	1286.36	Right Posterior Insula	E‐stim		2460.87	1874.05	1583.23
	Sour			3564.77	2875.49		Sour			2591.93	1986.01
	Visual				1163.97		Visual				1876.74
	Water						Water				

		L. VAI	L. DAI	L. PI	R. VAI	R. DAI	R. PI
E‐stim	L. VAI		3141.30	2686.46	1963.16	2939.86	2766.99
	L. DAI			1739.76	4483.43	2787.04	1284.48
	L. PI				5329.79	3368.74	631.41
	R. VAI					3576.92	5205.63
	R. DAI						2939.10
	R. PI						
Sour	L. VAI		2716.79	3829.89	1269.57	3816.65	4524.00
	L. DAI			1978.09	3604.21	4828.98	4878.66
	L. PI				3624.06	2910.31	2264.36
	R. VAI					2497.45	3228.80
	R. DAI						3350.59
	R. PI						
Visual	L. VAI		3862.98	3547.02	2286.79	3703.38	4214.58
	L. DAI			798.19	5255.07	3546.38	1354.24
	L. PI				5171.98	4983.16	926.62
	R. VAI					2546.01	4426.06
	R. DAI						2731.44
	R. PI						
Water	L. VAI		1666.67	2508.63	2422.38	3870.21	2551.79
	L. DAI			3492.75	3322.44	2050.63	3295.61
	L. PI				3301.94	4927.21	888.58
	R. VAI					2419.45	3859.23
	R. DAI						5592.67
	R. PI						
Uniform	E‐stim	3161.63	3275.64	3865.32	4092.28	2427.34	3541.91
	Sour	3341.14	4995.55	3794.68	2376.53	2453.71	4819.70
	Visual	4908.68	4528.56	4204.54	2858.53	3210.85	3434.88
	Water	2686.09	2855.98	3250.39	3455.75	3705.96	3286.54
Laterality	E‐stim	3626.20	3474.29	3060.30	1249.06	3485.63	3204.54
	Sour	2423.58	822.01	2072.77	3310.44	5395.07	3983.45
	Visual	4124.74	4631.07	3815.12	2374.23	5069.51	4005.87
	Water	3326.74	3051.58	4561.79	3999.08	3139.46	3807.10

L., Left; R., Right; VAI, ventral anterior insular seed; DAI, dorsal anterior insular seed; PI, posterior insular seed; Uniform, tests the spatial patterns against a uniform distribution across regions.

Sensitization was evident in this insular region with increasing functional connectivity during water swallows in frontal and parietal regions (M1, S1, IFG triangularis, SMA) and during the visual biofeedback condition in frontal regions (M1, IFG triangularis and opercularis). Habituation was found where functional connectivity gradually decreased in the sour condition in multiple regions (S1, IPG, ACC, and insula) as well as the e‐stim condition (M1, rolandic operculum).

#### Dorsal anterior insula

The dorsal anterior insula had the least amount of functional connectivity changes during swallowing. At our threshold (cluster corrected *P* < 0.05), the dorsal anterior insula was not associated with significant functional connectivity changes when swallowing water or when swallowing with e‐stim. The visual biofeedback and the sour conditions were associated with minimal connectivity increases within sensorimotor areas of the frontal and parietal lobes and only found with the left dorsal anterior insula. The analysis of the spatial distribution of connectivity increases revealed that, indeed, the left and right dorsal anterior regions have different patterns of functional connectivity increases and that the patterns are lateralized to the ipsilateral hemisphere (Figs. 4, 5, Tables 3 and 4). Additionally, each task pattern was significantly different (Figs. 5, 7, Table 4).

No sensitization effects were found for the dorsal anterior insula. However, during sour swallows, functional connectivity between the dorsal anterior insula and M1, IFG opercularis and orbitalis, SMA, S1, IPG, SMG, the rolandic operculum and insula decreased over 20 consecutive trials (habituation). Habituation in functional connectivity was also observed during water swallows, with decreasing connectivity between the dorsal anterior insula and the rolandic operculum, insula and SMA. Several of these effects were significantly different between conditions (Tables 5–10).

**Table 5.** Significant left ventral anterior insular PPI effects during oropharyngeal swallowing.

Contrast	Cluster Size	Peak T‐statistic	Peak X, Y, Z	Region
Sour > Baseline	63	6.51	−28, 22, −12	L. Insula
		3.37	−36, 14, −7	L. Insula
Visual > Baseline	63	6.92	−26, −36, −6	L. Hippocampus
	194	6.89	−2, 20, 52	L. Supplementary Motor Area
	1009	6.77	30, −56, 46	R. Angular Gyrus
		6.45	36, −40, 44	R. Supramarginal Gyrus
		5.29	42, −52, 54	R. Inferior Parietal Lobule
		5.07	50, −31, 59	R. Postcentral Gyrus
		4.83	36, −48, 64	R. Postcentral Gyrus
		4.72	34, −48, 52	R. Inferior Parietal Lobule
		4.34	50, −44, 44	R. Supramarginal Gyrus
		4.25	42, −50, 46	R. Inferior Parietal Lobule
		4.18	50, −42, 54	R. Inferior Parietal Lobule
		3.79	50, −27, 45	R. Postcentral Gyrus
	1097	6.45	−46, −46, 50	L. Inferior Parietal Lobule
		6.21	−50, −32, 50	L. Postcentral Gyrus
		6.05	−40, −54, 52	L. Inferior Parietal Lobule
		6.04	−32, −50, 38	L. Inferior Parietal Lobule
		5.66	−40, −36, 44	L. Postcentral Gyrus
		4.28	−40, −52, 40	L. Inferior Parietal Lobule
		4.14	−26, −50, 46	L. Inferior Parietal Lobule
		4.11	−48, −40, 56	L. Inferior Parietal Lobule
		4.10	−27, −57, 50	L. Inferior Parietal Lobule
		3.96	−46, −30, 36	L. Inferior Parietal Lobule
		3.88	−52, −32, 42	L. Inferior Parietal Lobule
		3.53	−36, −60, 40	L. Angular Gyrus
		3.44	−52, −16, 41	L. Postcentral Gyrus
		3.14	−54, −22, 50	L. Postcentral Gyrus
	221	6.26	−42, −18, 60	L. Precentral Gyrus
		5.22	−28, −28, 66	L. Precentral Gyrus
		3.15	−32, −22, 70	L. Precentral Gyrus
	68	5.41	44, 20, −4	R. Insula
		4.06	46, 18, −14	R. Inferior Frontal Gyrus ‐ Orbital Part
	71	5.28	46, −21, 36	R. Postcentral Gyrus
	159	5.20	−30, −40, 66	L. Postcentral Gyrus
		4.12	−32, −42, 58	L. Postcentral Gyrus
		3.69	−18, −44, 70	L. Postcentral Gyrus
	57	4.66	−31, 21, −13	L. Inferior Frontal Gyrus ‐ Orbital Part
	54	4.19	−59, −3, 11	L. Rolandic Operculum
		3.04	−60, 6, 8	L. Inferior Frontal Operculum
	56	4.11	48, 10, 38	R. Precentral Gyrus
		3.72	50, 4, 32	R. Precentral Gyrus
		3.34	50, 20, 38	R. Inferior Frontal Operculum
	72	4.11	28, −36, −4	R. Hippocampus
		4.08	22, −32, −12	R. Parahippocampal Gyrus
		3.95	20, −40, −6	R. Parahippocampal Gyrus
	97	3.77	46, 24, 23	R. Inferior Frontal Gyrus ‐ Pars triangularis
		3.65	42, 16, 24	R. Inferior Frontal Gyrus ‐ Pars triangularis
		3.19	46, 30, 30	R. Inferior Frontal Gyrus ‐ Pars triangularis
Water > Baseline	162	8.42	−48, 6, 30	L. Precentral Gyrus
		3.96	−52, 14, 21	L. Inferior Frontal Operculum
		3.06	−42, 16, 34	L. Inferior Frontal Operculum
	73	5.86	18, −36, 62	R. Postcentral Gyrus
		4.20	28, −30, 62	R. Postcentral Gyrus
	170	4.96	−46, 24, −8	L. Inferior Frontal Gyrus ‐ Orbital Part
		4.10	−38, 28, −8	L. Inferior Frontal Gyrus ‐ Orbital Part
		3.90	−38, 20, −4	L. Insula
		2.98	−54, 20, 2	L. Inferior Frontal Gyrus ‐ Pars triangularis
	52	4.59	56, 32, 6	R. Inferior Frontal Gyrus ‐ Pars triangularis
	58	4.28	−42, −22, 59	L. Postcentral Gyrus
	59	4.08	−22, −35, 63	L. Postcentral Gyrus
		3.81	−24, −40, 56	L. Postcentral Gyrus
Visual > Estim	687	5.49	−46, −54, 48	L. Inferior Parietal Lobule
		3.81	−40, −64, 46	L. Angular Gyrus
		3.68	−30, −50, 36	L. Angular Gyrus
		3.13	−29, −58, 44	L. Inferior Parietal Lobule
	996	4.96	46, −52, 52	R. Inferior Parietal Lobule
		4.51	44, −58, 47	R. Inferior Parietal Lobule
		4.21	48, −42, 40	R. Supramarginal Gyrus
		3.62	32, −58, 44	R. Angular Gyrus
		3.28	40, −68, 40	R. Angular Gyrus
		3.18	32, −46, 38	R. Angular Gyrus
		3.16	34, −48, 54	R. Inferior Parietal Lobule
		2.95	36, −40, 42	R. Supramarginal Gyrus
		2.94	30, −68, 48	R. Angular Gyrus
	289	4.43	50, 30, 26	R. Inferior Frontal Gyrus ‐ Pars triangularis
		4.08	42, 18, 22	R. Inferior Frontal Gyrus ‐ Pars triangularis
		3.80	46, 12, 36	R. Inferior Frontal Operculum
		3.66	52, 20, 34	R. Inferior Frontal Operculum
		3.06	44, 8, 28	R. Inferior Frontal Operculum
	60	4.38	28, −18, −18	R. Hippocampus
		2.76	20, −16, −20	R. Parahippocampal Gyrus
	56	3.98	25, −33, −7	R. Parahippocampal Gyrus
	52	3.92	−2, 22, 54	L. Supplementary Motor Area
	66	3.67	−28, −38, 64	L. Postcentral Gyrus
		3.64	−22, −42, 68	L. Postcentral Gyrus
Visual > Sour	697	4.48	47, −52, 43	R. Inferior Parietal Lobule
		4.38	48, −48, 52	R. Inferior Parietal Lobule
		3.83	42, −38, 40	R. Supramarginal Gyrus
		3.51	58, −42, 42	R. Supramarginal Gyrus
		3.42	32, −56, 42	R. Angular Gyrus
		3.19	38, −60, 52	R. Angular Gyrus
	161	4.14	52, 22, 34	R. Inferior Frontal Operculum
		4.03	48, 30, 30	R. Inferior Frontal Gyrus ‐ Pars triangularis
		3.74	52, 12, 40	R. Precentral Gyrus
	235	3.97	−40, −52, 54	L. Inferior Parietal Lobule
		3.52	−48, −54, 48	L. Inferior Parietal Lobule
		3.18	−34, −48, 44	L. Inferior Parietal Lobule
	78	3.89	−28, −28, 66	L. Precentral Gyrus
		3.20	−20, −30, 66	L. Postcentral Gyrus
Visual > Water	119	3.65	49, −43, 44	R. Supramarginal Gyrus
Water > Estim	203	4.39	−46, −54, 46	L. Inferior Parietal Lobule
		3.96	−42, −50, 32	L. Angular Gyrus
	108	4.06	−45, 17, 34	L. Inferior Frontal Operculum
		3.70	−50, 8, 34	L. Precentral Gyrus
	66	4.03	58, 30, 12	R. Inferior Frontal Gyrus ‐ Pars triangularis
		3.05	54, 40, 6	R. Inferior Frontal Gyrus ‐ Pars triangularis
	87	3.49	−38, 2, 26	L. Inferior Frontal Operculum
		3.48	−42, −4, 32	L. Precentral Gyrus
	56	3.37	46, 28, 28	R. Inferior Frontal Gyrus ‐ Pars triangularis
Water > Sour	54	4.14	−50, 10, 34	L. Precentral Gyrus
	79	3.90	−48, 22, −6	L. Inferior Frontal Gyrus ‐ Orbital Part
		3.53	−52, 14, 0	L. Inferior Frontal Operculum
		3.08	−40, 24, −4	L. Inferior Frontal Gyrus ‐ Orbital Part
		2.77	−54, 24, 6	L. Inferior Frontal Gyrus ‐ Pars triangularis
Habituation				
Estim	79	4.37	−48, 0, 20	L. Precentral Gyrus
		3.72	−50, 6, 26	L. Precentral Gyrus
		3.54	−50, 12, 32	L. Precentral Gyrus
		3.17	−50, −2, 10	L. Rolandic Operculum
Sour	91	4.75	39, 12, −2	R. Insula
		3.78	32, 16, −10	R. Insula
	60	4.61	−2, 30, 14	L. Anterior Cingulate Cortex
		3.85	4, 26, 18	R. Anterior Cingulate Cortex
		3.66	−4, 24, 19	L. Anterior Cingulate Cortex
	222	4.48	−52, −18, 34	L. Postcentral Gyrus
		4.46	−45, −25, 38	L. Inferior Parietal Lobule
		4.01	−34, −30, 38	L. Inferior Parietal Lobule
		4.00	−50, −10, 24	L. Postcentral Gyrus
		3.30	−34, −38, 42	L. Inferior Parietal Lobule
		3.11	−44, −34, 40	L. Inferior Parietal Lobule
		3.09	−58, −4, 24	L. Postcentral Gyrus
Sensitization				
Estim	93	5.40	49, −8, 37	R. Precentral Gyrus
		4.52	40, −17, 43	R. Precentral Gyrus
	83	4.20	−33, −35, 42	L. Postcentral Gyrus
Visual	86	6.02	−48, −2, 20	L. Precentral Gyrus
		3.72	−44, 8, 28	L. Inferior Frontal Operculum
	99	4.52	−47, 30, 8	L. Inferior Frontal Gyrus ‐ Pars triangularis
Water	601	6.59	9, −26, 61	R. Precentral Gyrus
		6.02	22, −26, 64	R. Precentral Gyrus
		5.09	36, −16, 50	R. Precentral Gyrus
		4.55	36, −22, 58	R. Precentral Gyrus
		4.27	46, −16, 56	R. Precentral Gyrus
		4.09	46, −26, 57	R. Postcentral Gyrus
		3.90	−7, −17, 56	L. Supplementary Motor Area
		3.63	30, −20, 68	R. Precentral Gyrus
		3.27	38, −26, 50	R. Postcentral Gyrus
	307	6.37	−22, −26, 64	L. Precentral Gyrus
		4.81	−24, −38, 62	L. Postcentral Gyrus
		4.39	−38, −36, 62	L. Postcentral Gyrus
		3.68	−36, −24, 52	L. Postcentral Gyrus
	63	5.71	36, −32, 62	R. Postcentral Gyrus
	148	4.79	22, −40, 68	R. Postcentral Gyrus
		4.19	14, −40, 66	R. Postcentral Gyrus
		3.65	24, −36, 60	R. Postcentral Gyrus
	61	4.72	34, −40, 54	R. Postcentral Gyrus
	55	4.19	−40, 20, 30	L. Inferior Frontal Gyrus ‐ Pars triangularis
Estim > Sour	206	5.28	−32, −35, 43	L. Inferior Parietal Lobule
		4.84	−36, −32, 36	L. Inferior Parietal Lobule
Sour > Estim	124	4.35	−38, −72, 40	L. Angular Gyrus
		3.40	−38, −62, 38	L. Angular Gyrus
Visual > Estim	275	5.66	−52, 14, 37	L. Precentral Gyrus
		3.87	−50, 4, 40	L. Precentral Gyrus
		3.29	−48, −2, 22	L. Precentral Gyrus
	230	5.17	−34, −74, 42	L. Inferior Parietal Lobule
		3.23	−34, −60, 38	L. Angular Gyrus
		3.22	−47, −56, 48	L. Inferior Parietal Lobule
		3.21	−38, −64, 32	L. Angular Gyrus
	70	3.85	44, −68, 38	R. Angular Gyrus
Visual > Sour	504	5.20	−52, 4, 40	L. Precentral Gyrus
		3.80	−50, −4, 22	L. Precentral Gyrus
		3.74	−53, −5, 30	L. Precentral Gyrus
		3.72	−62, −16, 16	L. Postcentral Gyrus
		3.64	−62, −2, 22	L. Postcentral Gyrus
		3.50	−60, −16, 34	L. Postcentral Gyrus
	57	4.36	−58, 20, 13	L. Inferior Frontal Gyrus ‐ Pars triangularis
	59	3.82	52, −2, 21	R. Rolandic Operculum
Water > Estim	615	5.44	−40, −65, 39	L. Angular Gyrus
		5.15	−36, −74, 42	L. Inferior Parietal Lobule
		4.72	−50, −66, 34	L. Angular Gyrus
		4.40	−48, −60, 44	L. Inferior Parietal Lobule
	204	5.39	−44, 18, 32	L. Inferior Frontal Operculum
		5.32	−52, 18, 34	L. Inferior Frontal Operculum
		4.35	−52, 12, 40	L. Precentral Gyrus
	669	5.17	36, −22, 58	R. Precentral Gyrus
		4.43	−8, −18, 56	L. Supplementary Motor Area
		4.26	30, −32, 64	R. Postcentral Gyrus
		4.03	26, −24, 60	R. Precentral Gyrus
		3.90	4, −22, 60	R. Supplementary Motor Area
		3.90	18, −26, 68	R. Precentral Gyrus
		3.85	22, −40, 68	R. Precentral Gyrus
		3.67	10, −23, 53	R. Supplementary Motor Area
		3.41	14, −40, 64	R. Postcentral Gyrus
		2.84	6, −18, 68	R. Supplementary Motor Area
	197	3.99	36, −68, 48	R. Angular Gyrus
		3.61	40, −67, 41	R. Angular Gyrus
		3.46	48, −60, 30	R. Angular Gyrus
		3.17	50, −60, 42	R. Angular Gyrus
	121	3.96	−21, −25, 62	L. Precentral Gyrus
		3.59	−30, −24, 58	L. Precentral Gyrus
	76	3.75	−34, −34, 62	L. Postcentral Gyrus
		3.56	−21, −39, 65	L. Postcentral Gyrus
Water > Sour	105	5.21	−44, 20, 34	L. Inferior Frontal Operculum
		3.03	−38, 14, 24	L. Inferior Frontal Gyrus ‐ Pars triangularis
	89	4.68	−56, 18, 16	L. Inferior Frontal Gyrus ‐ Pars triangularis
	572	4.54	−32, −23, 59	L. Precentral Gyrus
		4.17	−20, −28, 62	L. Postcentral Gyrus
		3.96	−52, −28, 48	L. Inferior Parietal Lobule
		3.85	−18, −18, 68	L. Precentral Gyrus
		3.78	−25, −37, 59	L. Postcentral Gyrus
		3.76	−34, −34, 62	L. Postcentral Gyrus
		3.26	−18, −38, 70	L. Postcentral Gyrus
		3.19	−46, −24, 52	L. Postcentral Gyrus
		3.03	−50, −22, 36	L. Inferior Parietal Lobule
		2.95	−49, −36, 47	L. Inferior Parietal Lobule
	478	4.17	34, −22, 55	R. Precentral Gyrus
		4.16	20, −27, 70	R. Precentral Gyrus
		3.83	24, −38, 69	R. Postcentral Gyrus
		3.82	40, −24, 50	R. Postcentral Gyrus
		3.79	32, −30, 66	R. Postcentral Gyrus
		3.61	28, −22, 62	R. Precentral Gyrus
		3.53	46, −16, 56	R. Precentral Gyrus
		3.50	38, −34, 62	R. Postcentral Gyrus
		3.41	18, −18, 68	R. Precentral Gyrus
		3.32	25, −46, 62	R. Postcentral Gyrus
		3.12	26, −36, 56	R. Postcentral Gyrus
		3.12	16, −40, 66	R. Postcentral Gyrus
	64	4.08	44, 16, 38	R. Inferior Frontal Operculum
		3.66	52, 10, 36	R. Precentral Gyrus
	80	3.89	46, −50, 28	R. Angular Gyrus
	77	3.66	6, −18, 66	R. Supplementary Motor Area
		3.39	−4, −18, 56	L. Supplementary Motor Area
	104	3.56	−51, −62, 37	L. Angular Gyrus
		3.04	−48, −56, 26	L. Angular Gyrus

Habituation is the inverse contrast of sensitization. Thus, condtion comparisons for sensitization could indicate decreased habituation effects in addition to increased sensitization effects. L., Left; R., Right.

**Table 6.** Significant right ventral anterior insular PPI effects during oropharyngeal swallowing.

Contrast	Cluster size	Peak T‐statistic	Peak X, Y, Z	Region
Sour > Baseline	58	6.13	32, 22, −12	R. Inferior Frontal Gyrus ‐ Orbital Part
Visual > Baseline	75	4.41	−26, −40, −2	L. Hippocampus
		4.31	−29, −34, −9	L. Hippocampus
		4.13	−18, −36, 1	L. Hippocampus
Visual > Estim	75	4.48	48, −2, 28	R. Precentral Gyrus
		3.98	42, 0, 38	R. Precentral Gyrus
	81	4.39	−32, −36, 64	L . Postcentral Gyrus
	145	4.36	−36, −20, 63	L. Precentral Gyrus
Visual > Water	69	4.52	−17, −29, −11	L. Parahippocampal Gyrus
		4.29	−16, −34, −2	L. Hippocampus
Water > Estim	157	4.14	−32, −22, 60	L. Precentral Gyrus
		3.86	−36, −14, 66	L. Precentral Gyrus
	55	3.71	−44, −28, 56	L. Postcentral Gyrus
	76	3.59	−26, −34, 66	L. Postcentral Gyrus
		3.30	−34, −34, 68	L. Postcentral Gyrus
Water > Sour	54	3.25	−28, −24, 56	L. Precentral Gyrus
		3.25	−32, −25, 66	L. Precentral Gyrus
Habituation	No Significant Effects			
Sensitization				
Sour > Estim	71	3.64	−48, −28, 50	L. Postcentral Gyrus
		3.03	−40, −26, 46	L. Postcentral Gyrus
Water > Estim	273	4.47	−42, −62, 42	L. Angular Gyrus
		4.11	−28, −72, 46	L. Inferior Parietal Lobule
	87	4.13	36, −64, 48	R. Angular Gyrus
Water > Sour	60	3.05	−52, −50, 38	L. Inferior Parietal Lobule
		3.01	−47, −57, 35	L. Angular Gyrus

Habituation is the inverse contrast of sensitization. Thus, condtion comparisons for sensitization could indicate decreased habituation effects in addition to increased sensitization effects. L., Left; R., Right.

**Table 7.** Significant left dorsal anterior insular PPI effects during oropharyngeal swallowing.

Contrast	Cluster size	Peak T‐statistic	Peak X, Y, Z	Region
Estim > Baseline	53	4.86	−36, 17, 19	L. Inferior Frontal Operculum
Visual > Baseline	229	4.74	48, −28, 38	R. Supramarginal Gyrus
		4.65	52, −20, 38	R. Postcentral Gyrus
		4.53	56, −8, 42	R. Precentral Gyrus
		3.37	61, −13, 31	R. Postcentral Gyrus
	129	4.72	−58, −8, 32	L. Postcentral Gyrus
		4.32	−55, −10, 42	L. Postcentral Gyrus
		3.22	−62, −2, 24	L. Postcentral Gyrus
	161	4.44	−56, −23, 36	L. Supramarginal Gyrus
		3.40	−48, −26, 44	L. Inferior Parietal Lobule
Estim > Sour	64	4.23	−40, 14, 28	L. Inferior Frontal Gyrus ‐ Pars triangularis
	205	4.05	−57, −36, 40	L. Inferior Parietal Lobule
		3.23	−60, −48, 38	L. Inferior Parietal Lobule
		2.83	−48, −42, 36	L. Inferior Parietal Lobule
	89	3.67	32, −36, 68	R. Postcentral Gyrus
		3.24	30, −38, 58	R. Postcentral Gyrus
		3.19	29, −29, 72	R. Postcentral Gyrus
		3.16	40, −32, 62	R. Postcentral Gyrus
	59	3.53	52, −26, 56	R. Postcentral Gyrus
Visual > Sour	985	4.36	−54, −28, 52	L. Postcentral Gyrus
		4.11	−42, −36, 48	L. Postcentral Gyrus
		3.88	−50, −26, 36	L. Inferior Parietal Lobule
		3.82	−54, −12, 50	L. Postcentral Gyrus
		3.64	−62, −36, 36	L. Supramarginal Gyrus
		3.52	−56, −34, 42	L. Inferior Parietal Lobule
		3.38	−50, −42, 54	L. Inferior Parietal Lobule
		3.38	−38, −40, 64	L. Postcentral Gyrus
		3.31	−58, −8, 38	L. Postcentral Gyrus
		3.24	−32, −32, 50	L. Postcentral Gyrus
		3.16	−58, −48, 40	L. Inferior Parietal Lobule
		2.85	−27, −38, 63	L. Postcentral Gyrus
	226	3.86	−54, 6, 40	L. Precentral Gyrus
		3.72	−52, 8, 28	L. Inferior Frontal Operculum
		3.32	−46, 14, 30	L. Inferior Frontal Gyrus ‐ Pars triangularis
		3.13	−59, 4, 18	L. Precentral Gyrus
		2.95	−50, 0, 18	L. Precentral Gyrus
	359	3.80	52, −24, 44	R. Postcentral Gyrus
		3.78	62, −16, 44	R. Postcentral Gyrus
		3.46	54, −12, 48	R. Precentral Gyrus
		3.14	52, −18, 38	R. Postcentral Gyrus
		3.04	52, −28, 56	R. Postcentral Gyrus
		2.90	56, −6, 40	R. Precentral Gyrus
	52	3.77	−10, 22, 60	L. Supplementary Motor Area
		3.26	0, 14, 62	L. Supplementary Motor Area
	278	3.68	−1, −4, 54	L. Supplementary Motor Area
		3.48	8, −8, 58	R. Supplementary Motor Area
		3.19	−8, 11, 46.5	L. Supplementary Motor Area
Habituation				
Sour	105	7.06	−40, 34, −12	L. Inferior Frontal Gyrus ‐ Orbital Part
		4.52	−30, 36, −12	L. Inferior Frontal Gyrus ‐ Orbital Part
		3.65	−40, 38, −4	L. Inferior Frontal Gyrus ‐ Orbital Part
	211	6.06	48, 20, −4	L. Inferior Frontal Gyrus ‐ Orbital Part
		3.31	48, −4, 0	R. Insula
	180	5.59	12, −22, 52	R. Supplementary Motor Area
		4.54	13, −23, 65	R. Supplementary Motor Area
		4.27	12, −12, 54	R. Supplementary Motor Area
		3.63	1, −13, 55	R. Supplementary Motor Area
	109	5.33	10, 1, 46	R. Supplementary Motor Area
		3.13	4, −2, 56	R. Supplementary Motor Area
	75	5.16	40, −18, 52	R. Precentral Gyrus
		3.91	48, −16, 50	R. Precentral Gyrus
	74	4.92	62, −24, 36	R. Supramarginal Gyrus
		3.70	57, −16, 41	R. Postcentral Gyrus
	58	4.90	10, 4, 66	R. Supplementary Motor Area
		4.26	16, 6, 60	R. Supplementary Motor Area
	243	4.73	−36, 0, −10	L. Insula
		4.54	−36, 18, −6	L. Insula
		4.30	−42, 8, −4	L. Insula
		3.04	−40, −4, 4	L. Insula
	81	4.26	47, 3, 42	R. Precentral Gyrus
	59	4.14	38, −10, −3	R. Insula
		3.66	38, −19, 1	R. Insula
	206	4.05	−64, −36, 24	L. Supramarginal Gyrus
		3.84	−60, −20, 28	L. Postcentral Gyrus
		3.80	−57, −35, 34	L. Supramarginal Gyrus
		3.53	−62, −28, 28	L. Supramarginal Gyrus
		3.35	−50, −32, 44	L. Inferior Parietal Lobule
Water	164	5.72	−46, 2, 12	L. Rolandic Operculum
		4.41	−54, 2, 4	L. Rolandic Operculum
		4.04	−54, 2, 12	L. Rolandic Operculum
		3.57	−46, 2, −2	L. Insula
	214	5.17	0, 4, 54	L. Supplementary Motor Area
		3.67	6, 10, 54	R. Supplementary Motor Area
		3.62	6, −2, 62	R. Supplementary Motor Area
		3.33	6, 8, 46	R. Supplementary Motor Area
		3.29	6, 16, 48	R. Supplementary Motor Area
Sensitization				
Estim > Water	137	4.82	−48, 4, 14	L. Rolandic Operculum
		3.08	−52, −2, 2	L. Rolandic Operculum
		2.98	−50, 6, 30	L. Precentral Gyrus
	233	4.43	1, 3, 57	L. Supplementary Motor Area
	61	4.00	60, −6, 40	R. Postcentral Gyrus
		2.98	56, −12, 48	R. Precentral Gyrus
	84	3.52	58, −8, 18	R. Rolandic Operculum
Visual > Estim	82	3.85	−52, −22, 38	L. Inferior Parietal Lobule
		3.14	−48, −20, 28	L. Postcentral Gyrus
		3.12	−44, −22, 44	L. Postcentral Gyrus
Visual > Sour	117	4.61	44, 4, 42	R. Precentral Gyrus
	53	4.44	30, −10, 54	R. Precentral Gyrus
		4.09	34, −10, 62	R. Precentral Gyrus
	1001	4.34	−64, −40, 32	L. Supramarginal Gyrus
		4.09	−42, −24, 44	L. Postcentral Gyrus
		3.97	−58, −18, 16	L. Postcentral Gyrus
		3.88	−50, −22, 40	L. Inferior Parietal Lobule
		3.71	−56, 2, 34	L. Precentral Gyrus
		3.69	−60, −6, 34	L. Postcentral Gyrus
		3.65	−52, −34, 44	L. Inferior Parietal Lobule
		3.55	−40, −36, 48	L. Postcentral Gyrus
		3.49	−42, −16, 40	L. Postcentral Gyrus
		3.47	−60, −26, 38	L. Supramarginal Gyrus
		3.42	−64, −6, 20	L. Postcentral Gyrus
		3.41	−54, −32, 36	L. Inferior Parietal Lobule
		3.34	−62, −20, 28	L. Postcentral Gyrus
		3.11	−56, −52, 36	L. Inferior Parietal Lobule
		3.06	−64, −30, 28	L. Supramarginal Gyrus
		3.05	−46, −24, 56	L. Postcentral Gyrus
		2.99	−54, −16, 50	L. Postcentral Gyrus
	202	4.26	50, 16, −2	R. Inferior Frontal Operculum
		3.74	52, 6, −2	R. Rolandic Operculum
	342	4.26	53, −15, 47	R. Precentral Gyrus
		4.06	38, −20, 52	R. Precentral Gyrus
		3.24	55, −24, 44	R. Postcentral Gyrus
		3.00	60, −24, 36	R. Supramarginal Gyrus
		2.93	32, −18, 44	R. Precentral Gyrus
	83	4.13	40, −6, −2	R. Insula
		2.80	48, −10, 4	R. Insula
	261	3.87	0, −12, 58	L. Supplementary Motor Area
		3.65	−2, 2, 48	L. Supplementary Motor Area
		3.62	2, −2, 56	R. Supplementary Motor Area
		3.51	−4, −6, 52	L. Supplementary Motor Area
		2.99	2, 11, 57	R. Supplementary Motor Area
	323	3.82	−40, 2, −12	L. Insula
		3.62	−32, 16, −11	L. Insula
		3.51	−36, −14, −4	L. Insula
		3.37	−45, −4, −3	L. Insula
		3.35	−44, 16, −6	L. Inferior Frontal Gyrus ‐ Orbital Part
		3.35	−36, −4, −8	L. Insula
		3.31	−42, 8, −4	L. Insula
		2.94	−30, 10, −16	L. Insula
	105	3.67	−46, 2, 50	L. Precentral Gyrus
		3.59	−44, 3, 39	L. Precentral Gyrus
		3.08	−38, −4, 44	L. Precentral Gyrus
	63	3.53	−33, −43, 63	L. Postcentral Gyrus
Visual > Water	375	5.10	0, 2, 56	L. Supplementary Motor Area
		3.77	4, 8, 50	R. Supplementary Motor Area
		2.80	−10, 12, 54	L. Supplementary Motor Area
	357	4.50	56, −12, 48	R. Precentral Gyrus
		4.03	60, −6, 40	R. Postcentral Gyrus
		3.54	58, 6, 36	R. Precentral Gyrus
		3.43	38, 4, 50	R. Precentral Gyrus
		3.43	54, −2, 46	R. Precentral Gyrus
		3.23	41, −5, 41	R. Precentral Gyrus
		3.12	44, −11, 35	R. Postcentral Gyrus
	201	4.45	62, −13, 17	R. Postcentral Gyrus
		2.81	54, −24, 18	R. Supramarginal Gyrus
	408	3.90	−47, 1, 9	L. Rolandic Operculum
		3.68	−44, −2, −4	L. Insula
		3.57	−36, −10, −1	
		3.47	−62, −8, 18	L. Postcentral Gyrus
		3.44	−60, −4, 32	L. Postcentral Gyrus
		3.31	−52, −1, 19	L. Precentral Gyrus
		3.24	−54, 2, 3	L. Rolandic Operculum
		3.21	−58, −4, 24	L. Postcentral Gyrus
	76	3.69	−41, −16, 42	L. Postcentral Gyrus
Water > Estim	92	3.89	62, 16, 10	L. Inferior Frontal Operculum
		3.19	60, 22, 18	L. Inferior Frontal Gyrus ‐ Pars triangularis
		3.16	52, 25, 4	L. Inferior Frontal Gyrus ‐ Pars triangularis
Water > Sour	163	3.90	−32, 18, −6	L. Insula
		3.71	−26, 22, 4	L. Insula
	170	3.72	51, 22, −1	L. Inferior Frontal Gyrus ‐ Pars triangularis
		3.65	48, 14, −2	R. Insula
		2.93	50, 30, −8	R. Inferior Frontal Gyrus ‐ Orbital Part
	103	3.70	−36, 42, −4	L. Inferior Frontal Gyrus ‐ Orbital Part
		3.53	−44, 40, −10	L. Inferior Frontal Gyrus ‐ Orbital Part
	54	3.54	37, 39, −7	R. Inferior Frontal Gyrus ‐ Orbital Part

Habituation is the inverse contrast of sensitization. Thus, condtion comparisons for sensitization could indicate decreased habituation effects in addition to increased sensitization effects. L., Left; R., Right.

**Table 8.** Significant right dorsal anterior insular PPI effects during oropharyngeal swallowing.

Contrast	Cluster size	Peak T‐statistic	Peak X, Y, Z	Region
Estim > Water	103	4.41	−54, −20, 22	L. Postcentral Gyrus
		3.52	−44, −20, 20	L. Rolandic Operculum
		3.03	−64, −20, 22	L. Postcentral Gyrus
	84	3.90	42, −2, 16	R. Rolandic Operculum
		3.07	50, −10, 20	R. Rolandic Operculum
		3.00	36, −4, 16	R. Insula
	64	3.58	14, −34, 62	R. Postcentral Gyrus
		3.50	24, −30, 64	R. Precentral Gyrus
		3.23	34, −26, 64	R. Precentral Gyrus
Visual > Estim	195	5.15	48, 4, 34	R. Precentral Gyrus
		4.47	54, 2, 26	R. Precentral Gyrus
Visual > Water	75	4.52	46, 4, 34	R. Precentral Gyrus
	94	4.35	−42, 2, 46	L. Precentral Gyrus
	66	4.14	24, −12, −12	R. Hippocampus
		2.84	16, −8, −14	R. Hippocampus
	70	4.10	42, −32, 58	R. Postcentral Gyrus
	61	3.93	30, −24, 64	R. Precentral Gyrus
		3.45	20, −22, 64	R. Precentral Gyrus
	491	3.72	46, −2, 16	R. Rolandic Operculum
		3.53	56, −14, 28	R. Supramarginal Gyrus
		3.51	64, −10, 24	R. Postcentral Gyrus
		3.50	61, −4, 11	R. Rolandic Operculum
		3.34	44, −12, 52	R. Precentral Gyrus
		3.34	50, −10, 23	R. Postcentral Gyrus
		3.13	50, −8, 37	R. Precentral Gyrus
		3.12	52, −20, 20	R. Rolandic Operculum
		3.10	60, −16, 14	R. Rolandic Operculum
		3.07	54, −8, 12	R. Rolandic Operculum
	54	3.46	−46, 16, 8	L. Inferior Frontal Gyrus ‐ Pars triangularis
	71	3.41	−40, −10, 58	L. Precentral Gyrus
		3.12	−39, −15, 37	L. Postentral Gyrus
Sour > Water	54	4.22	−42, 2, 46	L. Precentral Gyrus
Habituation				
Sour	138	6.62	−46, −2, 24	L. Precentral Gyrus
		4.12	−46, 4, 40	L. Precentral Gyrus
		3.82	−52, −13, 25	L. Postcentral Gyrus
	310	5.81	56, 14, 4	R. Inferior Frontal Operculum
		5.15	48, 14, −2	R. Insula
		4.22	43, −2, 10	R. Rolandic Operculum
		3.85	42, −2, 0	R. Insula
		3.13	42, 8, 6	R. Insula
	277	5.46	−35, −30, 19	L. Rolandic Operculum
		5.07	−44, −10, 10	L. Rolandic Operculum
		4.64	−38, −18, 16	L. Rolandic Operculum
	100	4.73	52, −18, 36	R. Postcentral Gyrus
		4.02	62, −18, 34	R. Postcentral Gyrus
	75	4.69	60, −44, 24	R. Supramarginal Gyrus
	72	4.48	−38, 12, 4	L. Insula
		4.04	−32, 14, −8	L. Insula
	61	4.21	46, −34, 24	R. Supramarginal Gyrus
	81	3.99	62, −22, 18	R. Supramarginal Gyrus
		3.74	66, −14, 20	R. Postcentral Gyrus
Sensitization				
Estim > Sour	103	4.22	−48, 2, 24	L. Precentral Gyrus
		3.73	−48, −2, 6	L. Rolandic Operculum
	66	3.95	−48, 8, 42	L. Precentral Gyrus
Visual > Estim	63	3.92	−36, 28, −10	L. Inferior Frontal Gyrus ‐ Orbital Part
Visual > Sour	143	4.88	48, −4, 4	R. Rolandic Operculum
	91	3.75	50, 12, 0	R. Inferior Frontal Operculum
	61	3.42	−42, −15, 10	L. Rolandic Operculum
		3.19	−38, −16, 0	L. Insula
		3.00	−34, −22, 12	L. Insula
Water > Estim	110	3.76	−52, −16, 26	L. Postcentral Gyrus
		3.15	−52, −14, 34	L. Postcentral Gyrus
		3.11	−52, −14, 44	L. Postcentral Gyrus
Water > Sour	61	3.84	−52, −16, 26	L. Postcentral Gyrus
	110	3.58	44, −22, 38	R. Postcentral Gyrus
		3.46	62, −20, 34	R. Postcentral Gyrus
	75	3.29	56, −50, 24	R. Angular Gyrus
		2.99	52, −49, 33	R. Angular Gyrus

Habituation is the inverse contrast of sensitization. Thus, condtion comparisons for sensitization could indicate decreased habituation effects in addition to increased sensitization effects. L., Left; R., Right.

**Table 9.** Significant left posterior insular PPI effects during oropharyngeal swallowing.

Contrast	Cluster size	Peak T‐statistic	Peak X, Y, Z	Region
Estim > Baseline	111	5.1	−54, −2, 2	L. Rolandic Operculum
		4.4	−32, 0, 12	L. Insula
		4.25	−36, −10, 16	L. Insula
		3.93	−44, −5, 9	L. Rolandic Operculum
	279	5.06	−56, −38, 34	L. Supramarginal Gyrus
		4.09	−59, −27, 37	L. Supramarginal Gyrus
		3.90	−57, −1, 28	L. Precentral Gyrus
		3.62	−58, −16, 38	L. Postcentral Gyrus
		3.51	−55, −9, 31	L. Postcentral Gyrus
	79	3.65	57, −7, 29	R. Postcentral Gyrus
		3.64	64, −4, 26	R. Postcentral Gyrus
Sour > Baseline	169	6.63	−54, −2, 2	L. Rolandic Operculum
		4.87	−58, 2, 10	L. Rolandic Operculum
		4.50	−52, −6, 20	L. Postcentral Gyrus
		3.94	−41, 0, 25	L. Precentral Gyrus
		3.36	−46, −10, 28	L. Postcentral Gyrus
		3.10	−62, 2, 18	L. Postcentral Gyrus
	186	6.19	−44, −4, 8	L. Rolandic Operculum
		5.59	−36, −6, 6	L. Insula
		3.74	−36, 4, 4	L. Insula
		3.27	−38, −10, −2	L. Insula
	70	5.65	62, 2, 16	
	56	5.23	56, −44, 26	R. Supramarginal Gyrus
	98	5.14	40, 28, 24	R. Inferior Frontral Gyrus ‐ Pars triangularis
		3.53	54, 22, 26	R. Inferior Frontral Gyrus ‐ Pars triangularis
		3.51	44, 18, 22	R. Inferior Frontral Gyrus ‐ Pars triangularis
		3.40	52, 18, 18	R. Inferior Frontral Gyrus ‐ Pars triangularis
	58	4.03	−58, −22, 28	L. Postcentral Gyrus
Visual > Baseline	1511	6.68	−54, 6, 2	L. Rolandic Operculum
		6.40	−56, 4, 26	L. Precentral Gyrus
		5.89	−60, −38, 36	L. Supramarginal Gyrus
		5.76	−58, −22, 33	L. Postcentral Gyrus
		5.67	−54, −2, 6	L. Rolandic Operculum
		5.23	−58, 3, 18	L. Postcentral Gyrus
		5.02	−50, −10, 30	L. Postcentral Gyrus
		4.99	−36, −8, 2	L. Insula
		4.95	−55, 1, 34	L. Precentral Gyrus
		4.56	−64, −6, 24	L. Postcentral Gyrus
		4.44	−38, 8, 4	L. Insula
		4.34	−44, −4, 12	L. Rolandic Operculum
		4.06	−58, −14, 22	L. Postcentral Gyrus
		3.96	−50, −26, 46	L. Inferior Parietal Lobule
		3.83	−45, 1, 24	L. Inferior Frontal Operculum
		3.71	−45, 2, 4	L. Insula
		3.65	−62, −24, 14	L. Supramarginal Gyrus
		3.45	−52, −36, 42	L. Inferior Parietal Lobule
		3.41	−50, −19, 19	L. Postcentral Gyrus
		3.18	−36, −8, 12	L. Insula
	116	6.66	−6, 10, 46	L. Supplementary Motor Area
		3.34	2, 10, 54	R. Supplementary Motor Area
	514	4.84	36, −16, 40	R. Precentral Gyrus
		4.80	44, −28, 42	R. Postcentral Gyrus
		4.63	66, −16, 24	R. Supramarginal Gyrus
		4.58	58, −10, 36	R. Postcentral Gyrus
		4.50	54, −22, 46	R. Postcentral Gyrus
		4.33	62, −4, 28	R. Postcentral Gyrus
		4.13	50, −12, 34	R. Postcentral Gyrus
		3.23	36, −30, 38	R. Postcentral Gyrus
	165	4.71	−50, 36, 10	L. Inferior Frontal Gyrus ‐ Pars triangularis
		3.87	−46, 46, 2	L. Inferior Frontal Gyrus ‐ Pars triangularis
	172	4.56	−34, −41, 38	L. Inferior Parietal Lobule
		3.91	−27, −48, 43	L. Inferior Parietal Lobule
		3.87	−37, −51, 39	L. Inferior Parietal Lobule
		3.84	−40, −34, 48	L. Postcentral Gyrus
	235	4.48	54, 1, 7	R. Rolandic Operculum
		4.12	60, −18, 12	R. Rolandic Operculum
		4.03	52, 1, 18	R. Rolandic Operculum
		3.88	48, −20, 14	R. Rolandic Operculum
		3.42	60, −8, 16	R. Rolandic Operculum
	100	4.41	−14, −6, 66	L. Supplementary Motor Area
		4.28	0, −5, 66	L. Supplementary Motor Area
		3.96	−4, −18, 58	L. Supplementary Motor Area
		3.27	2, −6, 56	R. Supplementary Motor Area
		3.09	−2, −14, 66	L. Supplementary Motor Area
	159	4.37	−36, −16, 64	L. Precentral Gyrus
		3.89	−26, −22, 67	L. Precentral Gyrus
		3.59	−40, −12, 58	L. Precentral Gyrus
		3.52	−26, −32, 72	L. Postcentral Gyrus
		3.42	−25, −36, 63	L. Postcentral Gyrus
		3.16	−22, −40, 56	L. Postcentral Gyrus
	127	4.11	62, −30, 34	R. Supramarginal Gyrus
		3.88	54, −36, 32	R. Supramarginal Gyrus
		3.78	62, −40, 24	R. Supramarginal Gyrus
		3.50	60, −40, 36	R. Supramarginal Gyrus
		3.10	50, −42, 36	R. Supramarginal Gyrus
Water > Baseline	442	7.26	−54, −2, 2	L. Rolandic Operculum
		6.58	−54, −6, 20	L. Postcentral Gyrus
		5.82	−42, −4, 10	L. Rolandic Operculum
		5.40	−37, −6, 2	L. Insula
		4.71	−54, 6, 2	L. Rolandic Operculum
		4.15	−52, −6, 32	L. Precentral Gyrus
	66	4.91	−50, 28, 2	L. Inferior Frontal Gyrus ‐ Pars triangularis
		3.09	−38, 32, 8	L. Inferior Frontal Gyrus ‐ Pars triangularis
		3.07	−50, 34, 10	L. Inferior Frontal Gyrus ‐ Pars triangularis
	297	4.83	62, −13, 13	R. Rolandic Operculum
		4.32	62, 2, 10	R. Rolandic Operculum
		4.09	56, 8, 0	R. Rolandic Operculum
		3.77	50, −6, 5	R. Rolandic Operculum
		3.73	56, 0, 20	R. Precentral Gyrus
		3.57	64, −2, 24	R. Postcentral Gyrus
		3.47	58, 6, 28	R. Precentral Gyrus
	75	4.66	36, −15, 9	R. Insula
		4.07	39, −3, 2	R. Insula
	114	4.22	−36, −21, 41	L. Postcentral Gyrus
		3.73	−28, −10, 48	L. Precentral Gyrus
		3.51	−36, −10, 47	L. Precentral Gyrus
	76	4.20	−58, −24, 20	L. Supramarginal Gyrus
		3.35	−52, −26, 14	L. Supramarginal Gyrus
Sour > Estim	51	3.65	32, −26, −8	R. Hippocampus
		3.31	32, −20, −24	R. Hippocampus
Viusal > Estim	68	4.47	−4, 12, 50	L. Supplementary Motor Area
Visual > Sour	56	3.90	−4, 10, 48	L. Supplementary Motor Area
	74	3.60	−60, −28, 28	L. Supramarginal Gyrus
		3.41	−64, −34, 34	L. Supramarginal Gyrus
		2.90	−56, −36, 26	L. Supramarginal Gyrus
Habituation				
Sour	72	5.37	−60, −1, 21	L. Postcentral Gyrus
		3.82	−50, −4, 16	L. Postcentral Gyrus
	62	5.14	−52, −32, 34	L. Supramarginal Gyrus
	120	4.59	−24, −30, −13	L. Parahippocampal Gyrus
		4.04	−16, −26, −8	L. Hippocampus
		3.94	−32, −22, −16	L. Hippocampus
		3.49	−24, −22, −14	L. Hippocampus
Visual	55	4.16	−46, 29, 18	L. Inferior Frontal Gyrus ‐ Pars triangularis
Sensitization				
Estim	67	6.39	−20, −38, −4	L. Parahippocampal Gyrus
		4.77	−28, −40, −2	L. Hippocampus
		4.19	−22, −36, 6	L. Hippocampus
Estim > Sour	88	4.08	18, −36, 9	R. Hippocampus
		3.19	33, −38, −7	R. Parahippocampus
		3.07	26, −36, 0	R. Hippocampus
	58	3.81	40, −14, 54	R. Precentral Gyrus
		3.75	42, −10, 64	R. Precentral Gyrus
Water > Sour	182	4.67	−41, −3, 38	L. Precentral Gyrus
		3.58	−40, −2, 56	L. Precentral Gyrus
	51	3.99	−33, −25, 50	L. Postcentral Gyrus
Water > Visual	81	3.83	−48, −70, 32	L. Angular Gyrus
		3.51	−38, −62, 26	L. Angular Gyrus

Habituation is the inverse contrast of sensitization. Thus, condtion comparisons for sensitization could indicate decreased habituation effects in addition to increased sensitization effects. L., Left; R., Right.

**Table 10.** Significant right posterior insular PPI effects during oropharyngeal swallowing.

Contrast	Cluster Size	Peak T‐statistic	Peak X, Y, Z	Region
Estim > Baseline	161	5.66	52, 2, 18	R. Precentral Gyrus
		3.89	54, −6, 10	R. Rolandic Operculum
		3.56	64, −4, 19	R. Postcentral Gyrus
		3.43	62, 6, 20	R. Precentral Gyrus
	56	4.16	−48, 12, 12	L. Inferior Frontal Operculum
		4.12	−49, 9, 3	L. Rolandic Operculum
Sour > Baseline	69	4.51	42, 6, 6	R. Insula
		3.68	50, 8, −6	R. Insula
		3.48	50, 0, −2	R. Insula
Visual > Baseline	627	6.29	−56, 6, 16	L. Inferior Frontal Operculum
		5.65	−46, 4, 8	
		4.11	−62, −2, 22	L. Postcentral Gyrus
		4.00	−52, 1, 34	L. Precentral Gyrus
		3.91	−42, 10, 22	L. Inferior Frontal Operculum
		3.80	−48, −10, 28	L. Postcentral Gyrus
		3.76	−54, 10, 28	L. Inferior Frontal Operculum
		3.64	−56, −7, 20	L. Postcentral Gyrus
		3.55	−54, 8, 1	
		3.48	−40, −14, 38	L. Postcentral Gyrus
		3.26	−38, 3, 26	L. Inferior Frontal Operculum
		3.19	−58, −8, 30	L. Postcentral Gyrus
	73	5.61	32, −26, 62	R. Precentral Gyrus
	274	5.48	−10, 38, 10	L. Anterior Cingulate Cortex
		5.24	6, 30, 24	R. Anterior Cingulate Cortex
		4.34	−2, 24, 18	L. Anterior Cingulate Cortex
		3.98	14, 34, 20	R. Anterior Cingulate Cortex
	291	5.38	−11, −4, 66	L. Supplementary Motor Area
		4.75	8, −4, 66	R. Supplementary Motor Area
		4.57	−4, −4, 56	L. Supplementary Motor Area
		4.01	0, −2, 66	L. Supplementary Motor Area
		3.91	9, −21, 68	R. Supplementary Motor Area
		3.67	16, −4, 64	R. Supplementary Motor Area
		3.21	18, −22, 64	R. Precentral Gyrus
	311	5.34	49, −4, 8	R. Rolandic Operculum
		4.88	54, 0, 16	R. Rolandic Operculum
		4.56	36, −10, 14	R. Insula
		3.66	58, 4, 30	R. Precentral Gyrus
		3.57	38, 2, 10	R. Insula
		3.06	46, 2, 2	R. Insula
	146	5.19	−31, −19, 67	L. Precentral Gyrus
		3.77	−40, −16, 58	L. Precentral Gyrus
	53	4.98	58, −17, 14	R. Rolandic Operculum
	91	4.59	8, 4, 46	R. Supplementary Motor Area
		4.17	−6, 10, 48	L. Supplementary Motor Area
		3.21	2, 12, 52	R. Supplementary Motor Area
	70	4.57	9, 18, 28	R. Anterior Cingulate Cortex
		3.63	−2, 14, 28	L. Anterior Cingulate Cortex
		3.49	−8, 20, 30	L. Anterior Cingulate Cortex
	92	4.54	−26, −36, 68	L. Postcentral Gyrus
		3.56	−36, −36, 62	L. Postcentral Gyrus
	345	4.51	−56, −36, 32	L. Supramarginal Gyrus
		3.95	−60, −26, 26	L. Supramarginal Gyrus
		3.80	−60, −22, 36	L. Supramarginal Gyrus
		3.61	−56, −18, 18	L. Postcentral Gyrus
		3.27	−60, −24, 14	L. Supramarginal Gyrus
	112	4.37	56, −10, 30	R. Postcentral Gyrus
		4.22	46, −12, 32	R. Postcentral Gyrus
		3.61	66, −14, 22	R. Postcentral Gyrus
	64	4.13	−29, −55, 35	L. Angular Gyrus
		3.90	−32, −46, 40	L. Inferior Parietal Lobule
Water > Baseline	264	6.79	−44, 32, 2	L. Inferior Frontal Gyrus ‐ Pars triangularis
		4.02	−48, 36, 22	L. Inferior Frontal Gyrus ‐ Pars triangularis
	540	5.34	54, −6, 8	R. Rolandic Operculum
		4.91	41, −5, 7	R. Insula
		4.88	64, −8, 10	R. Rolandic Operculum
		4.82	60, 0, 12	R. Rolandic Operculum
		4.62	55, 1, 2	R. Rolandic Operculum
		4.20	58, −4, 22	R. Postcentral Gyrus
		4.11	66, −8, 18	R. Postcentral Gyrus
		3.78	41, −8, −2	R. Insula
		3.68	49, −24, 16	R. Rolandic Operculum
		3.32	62, −27, 21	R. Supramarginal Gyrus
		3.14	48, −10, 4	R. Insula
		3.06	40, 0, −6	R. Insula
		2.95	58, −16, 24	R. Supramarginal Gyrus
	68	4.95	16, −24, 70	R. Precentral Gyrus
		3.82	10, −24, 64	R. Supplementary Motor Area
		3.31	24, −22, 60	R. Precentral Gyrus
	107	4.87	−56, −8, 8	L. Rolandic Operculum
		4.73	−48, −16, 14	L. Rolandic Operculum
		3.39	−56, −6, 20	L. Postcentral Gyrus
	77	4.85	47, −20, 56	R. Postcentral Gyrus
	138	4.77	−48, −34, 54	L. Precentral Gyrus
		4.13	−36, −36, 66	L. Precentral Gyrus
		3.84	−24, −36, 68	L. Precentral Gyrus
	68	4.74	56, 28, 10	R. Inferior Frontal Gyrus ‐ Pars triangularis
		3.60	48, 30, 5	R. Inferior Frontal Gyrus ‐ Pars triangularis
	64	4.27	−32, 14, 6	L. Insula
	96	4.15	37, −36, 62	R. Postcentral Gyrus
		3.93	28, −34, 62	R. Postcentral Gyrus
		3.71	22, −36, 68	R. Postcentral Gyrus
		3.69	32, −30, 56	R. Postcentral Gyrus
Estim > Sour	91	4.24	−34, 16, 26	L. Inferior Frontal Operculum
Visual > Sour	83	4.59	38, −32, −12	R. Hippocampus
		3.82	30, −36, −4	R. Hippocampus
	65	3.88	−26, −34, −10	L. Parahippocampal Gyrus
		3.58	−22, −32, −18	L. Parahippocampal Gyrus
		3.44	−14, −34, −12	L. Parahippocampal Gyrus
	86	3.55	−44, 14, 28	L. Inferior Frontal Gyrus ‐ Pars triangularis
Water > Estim	80	3.46	−48, 34, 5	L. Inferior Frontal Gyrus ‐ Pars triangularis
		3.38	−50, 24, 2	L. Inferior Frontal Gyrus ‐ Pars triangularis
Water > Sour	145	4.20	50, −12, 56	R. Precentral Gyrus
		3.74	52, −22, 56	R. Postcentral Gyrus
		3.48	58, −16, 50	R. Postcentral Gyrus
		3.39	52, −6, 50	R. Precentral Gyrus
		3.15	44, −22, 46	R. Postcentral Gyrus
		3.06	40, −34, 66	R. Postcentral Gyrus
	130	3.77	−46, 28, −4	L. Inferior Frontal Gyrus ‐ Orbital Part
		3.52	−43, 34, 5	L. Inferior Frontal Gyrus ‐ Pars triangularis
	87	3.35	−49, 16, 31	L. Inferior Frontal Gyrus ‐ Pars triangularis
		2.76	−38, 20, 26	L. Inferior Frontal Gyrus ‐ Pars triangularis
Habituation	No Significant Effects			
Sensitization				
Visual	112	5.28	−38, −2, 42	L. Precentral Gyrus
		5.03	−36, −2, 54	L. Precentral Gyrus
		3.34	−32, −2, 64	L. Precentral Gyrus
	73	4.19	−8, 6, 48	L. Supplementary Motor Area
		3.96	6, 7, 49	R. Supplementary Motor Area
		2.97	−10, −2, 46	L. Supplementary Motor Area
Water	73	7.90	44, −56, 30	R. Angular Gyrus
		4.35	42, −62, 22	R. Angular Gyrus
	63	5.66	−48, −50, 24	L. Supramarginal Gyrus
Visual > Estim	91	4.24	−36, −4, 42	L. Precentral Gyrus
Visual > Sour	187	4.86	−24, −20, −14	L. Hippocampus
		3.79	−24, −12, −20	L. Hippocampus
		3.69	−32, −22, −14	L. Hippocampus
		3.28	−26, −32, −14	L. Parahippocampal Gyrus
		3.06	−34, −32, −12	L. Hippocampus
		2.93	−18, −4, −20	L. Hippocampus
	59	4.25	−38, −4, 40	L. Precentral Gyrus
		2.95	−39, −2, 55	L. Precentral Gyrus
	71	4.13	26, −20, −12	R. Hippocampus
		3.59	20, −26, −14	R. Parahippocampal Gyrus
Water > Sour	53	4.24	4, 46, 26	R. Anterior Cingulate Cortex
		3.09	−1, 48, 16	L. Anterior Cingulate Cortex
		2.77	2, 40, 14	L. Anterior Cingulate Cortex
	62	3.82	23, −25, −16	R. Parahippocampal Gyrus

Habituation is the inverse contrast of sensitization. Thus, condtion comparisons for sensitization could indicate decreased habituation effects in addition to increased sensitization effects. L., Left; R., Right.

#### Posterior insula/rolandic operculum

The posterior insular had the greatest amount of significant functional connectivity increases overall (Table 1) at a cluster corrected threshold of *P* < 0.05. The global laterality index was left lateralized for e‐stim and sour swallows (Fig. 4, Table 3). The left laterality index had functional connectivity increases with the left hemisphere for all conditions and the right laterality index had connectivity increases with the right hemisphere for e‐stim and sour swallows. The other conditions were not significantly lateralized for the right posterior insula/rolandic operculum region. As in the ventral anterior insula, other than the visual biofeedback condition (right insula to left hemisphere), connectivity increases were generally ipsilateral. The analysis of the spatial distribution of connectivity increases revealed that the left and right posterior regions have different patterns of functional connectivity increases (Table 4); however, as can be seen in Figs. 5 and 7, are not as different as the anterior regions. The patterns are primarily lateralized to the left hemisphere (Table 3). As in the other regions, each task pattern was significantly different (Table 4).

Connectivity gradually increased with repeated swallows between the posterior insula and frontal areas during the visual task (M1, SMA) and in the water task in parietal regions (SMG and angular gyrus). Gradually decreasing functional connectivity was found between the left posterior insula and IFG triangularis for the visual task. The sour condition was associated with decreasing connectivity between the left posterior insula and S1, SMG, hippocampus and parahippocampus. A number of these effects were significantly different between conditions (Tables 5–10).

## Discussion

The goal of this study was to investigate bilateral increases in functional connectivity of the posterior, dorsal anterior, ventral anterior insular regions during four volitional swallowing tasks. There are three main findings from this study. First, the posterior insula/rolandic operculum had the largest and most clusters of functional connectivity among insular regions based on set‐level *P* ‐values, but the ventral anterior insula was functionally connected to a more diverse array of cortical regions based on the spatial distribution plots (Figs. 5, 7, Table 6). This diverse pattern demonstrates that influence of the ventral anterior insula is more extensive, but the posterior insula/rolandic operculum has a more directed influence during swallowing (e.g., changes in functional connectivity are localized to fewer brain regions, Fig. 4). Second, visual biofeedback was associated with the most functional connectivity increases between each insular region and distant cortical regions. Third, connectivity increases during swallowing are lateralized to the left hemisphere (Table 3).

Differences in connectivity between insular sub‐divisions and swallowing condition indicate that the different sub‐divisions have different functional roles during swallowing and that the contextual cues (e.g., taste and vision) modulate how the brain processes each swallow.

### Condition‐specific effects

The visual biofeedback task had the greatest amount of functional connectivity increases compared to sour swallowing, water swallowing, and swallowing with e‐stim across the insula. This is not surprising considering the insula's interoceptive properties and the multimodal sensory nature of the visual biofeedback task (includes visual and general oral sensation). When attending to interoceptive sensations (i.e., thirst, air hunger, heartbeat, or gastrointestinal distension), insular activation increases (Craig 2002; Kurth et al. 2010). Furthermore, the insula is involved in the neural network for “multiple demand” for focal attention to salient stimuli (Dosenbach et al. 2007; Nelson et al. 2010; Simmons et al. 2012). Highlighting the role of visual feedback on the insula, Caria et al. (2007) showed that individuals could increase BOLD‐magnitude in the anterior insula bilaterally using visual feedback‐specific cognitive training strategies with real‐time fMRI (Caria et al. 2007). Our visual biofeedback task combines both attention and salient afferent experiences, resulting in an interoceptive experience, which could explain why it elicited greater functional connectivity increases overall.

The sour condition was similar to visual biofeedback in that it was associated with increases in all three insular regions, but with far less intensity. This suggests that these two swallowing experiences (taste and visual) require processing from more diverse regions than swallowing water alone or water with e‐stim. This could be because water and water plus e‐stim both primarily activate general sensory afferents (pain and touch), as opposed to special sensory afferents for taste and vision, which is involved in the sour and visual biofeedback tasks.

As with the functional neural activation (Humbert et al. 2012), the e‐stim condition was associated with the least amount of connectivity increases. However, our findings cannot rule out the possibility of neural facilitation with repeated cutaneous electrical stimulation (Gallas et al. 2007; Doeltgen et al. 2010). Differences between our findings and others’ of e‐stim effects could be due to methodology (neural stimulation vs. neuroimaging – functional connectivity metric) or the stimulation protocol or stimulation locations.

### Insular regions

The insula is among the most highly integrated cortical regions of the brain both anatomically and functionally (Augustine 1996; Kurth et al. 2010). It is involved in cognitive, social–emotional, gustatory, and sensorimotor functions, among many others (Kurth et al. 2010). Its involvement in swallowing behavior in both normal and abnormal swallowing is well‐established, although the particular insular regions thought to be most important have been somewhat inconsistent among reports (Martin et al. 2001; Ludlow et al. 2007; Humbert et al. 2009, 2010; Riecker et al. 2009; Soros et al. 2011). Our findings suggest that the insula is both consistently and broadly involved in swallowing, with strongest dynamic functional connectivity from the posterior and ventral anterior aspects.

#### Posterior insula/rolandic operculum

The posterior insula/rolandic operculum is thought to have a more “local” pattern of connectivity to the sensorimotor and posterior cingulate cortex (Cerliani et al. 2012). The midposterior insula, in particular, may be most connected to premotor and sensorimotor areas and motor planning areas such as the SMA (Cauda et al. 2011; Deen et al. 2011). Functionally, it is associated with a range of sensory experiences from pleasant to neutral to unpleasant (Hua et al. 2008). Sensorimotor processing abilities were shown with electrical stimulation to the posterior insula, which elicited bodily movement (Showers and Lauer 1961) and the urge to move (Penfield and Faulk 1955). More specific to swallowing, the posterior insula/rolandic operculum is associated with sensations in the mouth and is thought to be part of the insular taste region (Rudenga et al. 2010; Small 2010). Soros et al. (2011) reported irregular or delayed swallowing with electrical stimulation of the right inferior posterior insula, but not the superior posterior insula.

Our results show greater functional connectivity increases between the posterior insula/rolandic operculum and many sensorimotor (primary, secondary, and integrative) cortical regions during our swallowing tasks compared to the two anterior insular regions. This contrasts our finding that more fMRI signal was found in the anterior insula compared to the posterior insula in these same participants for the same swallowing tasks (Humbert et al., 2012). This difference highlights a key aspect of psychophysiological interactions, specifically the ability to investigate how information is conveyed or relayed between brain regions. In the context of this study, anterior insular regions might drive swallowing, but the posterior insula/rolandic operculum region might modulate swallowing by its direct, specific connections. Furthermore, the similarity between connectivity profiles of the left and right regions could indicate a more central role of the posterior insula in monitoring behavior and feedback. Consistent with this notion was the finding that the e‐stim condition – possibly an unpleasant sensation – had more connectivity between the posterior insula and other cortical regions. Left fMRI lateralization of swallowing tasks has been reported (Malandraki et al. 2010). In this study, we also observed that more of the functional connectivity increases were in the left hemisphere furthering the notion that this area modulates swallowing based on sensory feedback.

#### Anterior insula

Connectivity with the anterior insula involves a more wide‐spread, highly connected pattern with other brain regions, including frontal, cingulate, parietal, cerebellar, and other anterior insula areas, compared to the posterior insula (Cauda et al. 2012). However, within the anterior insula, the dorsal and ventral components have anatomical and functional distinctions (Mesulam and Mufson 1982a, b; Kurth et al. 2010; Cauda et al. 2011; Deen et al. 2011; Cerliani et al. 2012). The dorsal anterior insula has strong connections to the ACC, prefrontal, opercular, and parietal regions for preferential functions such attention and processing. On the other hand, the ventral region has connections with the limbic and paralimbic systems (hippocampus, parahippocampus, ACC, entorhinal cortex, peri amygdaloid, temporal pole, and orbitofrontal cortex) for intense affective and emotional experiences. Daniels and Foundas (1997) reported that patients with lesions to the anterior insula had pharyngeal dysphagia. Riecker et al. (2009) later specified the ventral anterior insula as an important site for dysphagia.

Our findings show that the ventral anterior insula was functionally connected to the most diverse array of cortical regions compared to either the posterior or the dorsal anterior insula, although clusters of connectivity were much smaller than the posterior insula. The ventral anterior region had greater functional connectivity increases with the distal parts of the limbic system, but neither anterior insular region had functional connectivity increases with the ACC. Rudenga et al. (2010) reported that the anterior ventral insula consistently responded to oral stimulation despite pleasantness of the tastant.

Although the dorsal anterior region is reportedly more connected with swallowing‐related areas such as the prefrontal, opercular, and parietal regions compared to the ventral component (Kurth et al. 2010; Cerliani et al. 2012), it had the least functional connectivity increases overall. Kurth et al. (2010) reported that the anterior dorsal insula was not involved in processing of somatosensation or motion, so it is possible that the dorsal anterior insula played a role in attention and processing during swallowing tasks. This could explain why the visual biofeedback task had more functional connectivity than other conditions for the dorsal anterior insula. Additionally, we do not have a measure of the intrinsic connectivity during repeated swallowing to know if the lack of increased functional connectivity was due to a baseline shift. This possibility cannot be excluded given the increased connections (Kurth et al. 2010; Cerliani et al. 2012).

### Laterality, Habituation, and Sensitization

We predicted left lateralization during our swallowing conditions, consistent with the findings from Lowell et al. (2012) and Babaei et al. (2013). Functional connectivity increases were lateralized to the left, primarily ipsilaterally between the insula and distal brain regions. This suggests that swallowing connectivity with insular involvement is preferential to left hemispheric neural networks across a variety of conditions. We also predicted no adaptation across the same swallowing condition. Contrarily, we found gradual changes in signal amplitude with repeated exposure to the same stimuli in each insular region for every swallowing condition except e‐stim, which was stable.

### gPPI Power

Recent work has suggested that the effect sizes for PPI analyses are moderate to large (Cisler et al. 2013). In light of this work, we feel that it is important to discuss key aspects to increasing the power in PPI analyses.

First, gPPI analyses have been shown to reduce false negatives and improve detection of true positives across a wide range of task parameters compared to earlier implementations of PPI (McLaren et al. 2012; Cisler et al. 2013). Both studies indicate that, as in this study, gPPI should be used as the analysis method. Second, event‐related PPI analyses should model the duration, as done in this study, of the underlying neural activity, rather than simply using a duration of 0 as is traditionally done in event‐related task studies. Finally, it is important to note that it is entirely possible for an area to have the same neural activity, but have the connectivity with a second region change dependent on the context or task.

## Conclusions

The literature on involvement of the insula and Rolandic operculum during swallowing has been both overlapping (consistently active overall), but somewhat unclear, in terms of hemispheric dominance and task specificity. Our results are aligned with reports about the insula's interconnectivity and extensive involvement in multisensory and cognitive tasks. We were able to elucidate the increased involvement of the posterior and ventral anterior regions in swallowing. Additionally, we have shown that visual biofeedback during swallowing further acts to modulate functional connectivity, with multimodal input (e.g., visual during swallowing) leading to increased functional connectivity. This investigation is the first attempt at parceling out insular regions, tasks, populations, and possible adaptation over consecutive trials. Future studies are needed to investigate each of these components in depth. Nevertheless, the study highlights the use of generalized psychophysiological interactions in furthering our understanding of the neural underpinnings of complex behaviors.

## Acknowledgments

The content is solely the responsibility of the authors and does not necessarily represent the official views of the National Institute On Deafness and Communication Disorders, National Institute On Aging, or the National Institutes of Health.

## Conflict of Interest

None declared.
